# Supplementary material for: Proteome-wide 3D structure prediction provides insights into the ancestral metabolism of ancient archaea and bacteria
Source: Nat Commun. 2022 Dec 21;13:7861. doi: 10.1038/s41467-022-35523-8 (PMC9772386; doi:10.1038/s41467-022-35523-8)
Supplement: Supplementary file 1 — Supplementary Information [file 41467_2022_35523_MOESM1_ESM.pdf]

Proteome-wide 3D structure prediction reveals distinct evolutionary histories of metabolic modules in ancient archaea and bacteria

Supplementary Information

Supplementary Note

Similar physiology between A501 and 3DAC in pH, salinity and pressure

Both strain A501 and strain 3DAC grew only under strictly anaerobic conditions, and performed as a piezophilic hyperthermophile and a piezophilic thermophile, respectively. The growth under different NaCl concentration between A501 and 3DAC are quite similar, with both 2.5% (w/v) NaCl as the optimal growth salinity, and with the range of 1.0-5.0% (w/v) and 1.0-4.5% (w/v), respectively. Similar senecio for the pH: both A501 and 3DAC have the optimal growth pH 7.0, and the range of pH 4-9 for A501 while pH 5.5-8.5 for 3DAC. The hydrostatic pressure range are also similar, 0.1-70 MPa (optimal pressure 0.1 MPa-30 MPa) for A501 while 0.1-80 MPa (optimal 20 MPa) for 3DAC.

Essential amino acids for A501 and 3DAC

Growth experiments on leave-one-out set of the amino acids revealed that among 20 amino acids, eight are essential for the growth of A501 while 11 for 3DAC, among which seven amino acids overlap between the two specifies (i.e. L-arginine, L-histidine, L-methionine, L-phenylalanine, L-tyrosine, L-tryptophan and L-threonine). There were seven amino acids overlap between A501 and 3DAC, including L-arginine, L-histidine, L-methionine, L-phenylalanine, L-tyrosine, L-tryptophan and L-threonine (**Supplementary Figure 1**). In the presence of amino acids, carbohydrates could be used as additional carbon sources (**Supplementary Figure 1**). Among them, abiotic synthesis of three aromatic amino acids (i.e., L-phenylalanine, L-tyrosine and L-tryptophan) and L-methionine is thermodynamically favored in hot and moderately reducing environments like the hydrothermal vents[1,2]. Whereas, L-threonine, L-arginine, and L-histidine have been proposed to produce by prebiotic routes[3], and the two cationic amino acids (i.e., L-arginine and L-histidine) were observed *in vitro* spontaneous oligomerization to give great yields of higher molecular weight oligomers[4]. These evidences imply that these essential amino acids for the growth of A501 and 3DAC can be formed in the environment through abiotic processes without the need of the internal biosynthesis.

Detailed description of the functional annotation

We used these different annotation tools depending on their different specialties to obtain potential functional prediction as much as possible. For example, EggNOG is general annotation tool, the transportDB and TCDB are specialized on the collection of transmembrane proteins, whereas KEGG and MetaCyc is better at annotating intracellular reactions. Then we use the manual curation according to the literature

reported biochemistry and physiological experiments to verify the annotation from these automatic approaches on public databases. For protein structures, 64% of A501 proteins have PDB supporting ortholog, and 61% for 3DAC proteins. For the functional annotation, in this study, we focus on the two major functional categories, i.e., the Biosynthesis and metabolism, and the Energy production and conversion. Among 201 protein pairs (both ortholog and non-ortholog) of A501 and 3DAC in these two categories, literature supporting annotations of protein pairs are 91 in total, covered almost all enzymes in the central carbon metabolism and respiration processes shown in Fig. 3A-B. There are only 40 protein pairs in these two functional categories were rely on predicted functions from the public database without either literature or PDB supporting.

Comparison of hydrogen bonds between protein pairs of A501 and 3DAC

We compared the hydrogen bonds per residue between protein pairs of A501 and 3DAC. The functions of the proteins with significant more hydrogen bonds in A501 are particularly concentrated in certain function categories that are related to biosynthesis, metabolism and biological processes (**Supplementary Data 1**). Whereas, number of hydrogen bonds of proteins in other function categories, i.e., energy production and conversion, transporter and signal transduction, cell motility, are highly comparable between A501 and 3DAC **(Supplementary Figure 3)**.

Different k’ values of E. coli and Tehei’s work

E. coli used in Tehei’s paper[5] is MRE600, while that used in our work is strain BL21(DE3). Various types of *E. coli* may cause the differences in effective force constants <k’> of biomacromolecules. Besides, *E. coli* used in our work is grown anaerobically at 37 ^{o}C, while *E. coli* used in Tehei’s paper is grown in aerobic environment at 37 ^{o}C. Different culture conditions may also affect the value of thermal resilience of biomacromolecules in *E. coli*. However, <k’> of *E. coli* obtained from our work (0.31 N/m) and Tehei’s paper (0.39 N/m) are both significantly lower than that of 3DAC (0.67 N/m) and A501 (0.78 N/m), which demonstrates the biomacromolecules in thermophilic/hyperthermophilic prokaryotes is stable at high temperatures. Moreover, we have added the error bar of the resilience estimated from our work. As can be seen in Fig. 1E, the error bar is smaller than the difference between the <k’> values measured in our work and in Tehei’s paper. We suspect this difference is real and it should result from the different bacterial strains and different culture conditions between the two works.

In this work, we compared the thermal resilience (<k’>) of biomacromolecules obtained from the A501, 3DAC, and *E. coli* grown at their respective physiological temperatures (85, 75, and 37 ^{o}C) to illustrate that biomacromolecules in A501 and 3DAC are structurally more stable than those in *E. coli*. We note that the comparison of these cells at their respective physiological conditions is of direct biological relevance, and that is why we did so. However, the value of <k’> of a cell could differ when the preparation condition changes. Here, we collected the <k’> values of biomacromolecules in *E.coli* grown in different conditions, including the anaerobic environment, in D_2O , in an unstressed condition, and in heat-shocked condition, which is 0.31, 0.19, 0.42, and 0.30

N/m[5,6], respectively. As a result, the resulting value when averaged over different preparation conditions is 0.31±0.09 N/m. Although the spread among different conditions is larger than that obtained from a single condition, one still can deduce that the resilience of biomolecules in *E. coli* is significantly smaller than those in 3DAC and A501 (0.67 and 0.78 N/m), i.e., the latter two are structurally more stable. Moreover, 3DAC and A501 grow extremely slowly at conditions other than the physiological temperature or even die in the aerobic culture, thereby the weight of cells cannot reach the requirement for dynamic neutron scattering measurements. Thus, we cannot do a similar statistical analysis on these two cells.

Discussion about investigate the prevalence of cis-peptide and incorrect amino acid chirality in AlphaFold predicted structures

For protein chirality, considering all amino acid should adopt a left-handed conformation, we find that AlphaFold has a low error rate on predicted structures (**Supplementary Figure 11.**). AlphaFold can achieve good accuracy (Right-handed Cα rate < 1.007‰ in A501 and < 2.117‰ in 3DAC) in most amino acids except cysteine. In cysteine chirality, AlphaFold have a much higher error rate (highest right-handed Cα rate 4.25% in A501 and 7.76% in 3DAC across all 5 models and before/after relax). In AlphaFold paper supplemental information page 36, they also mentioned that "AlphaFold can produce almost exactly the chiral pair for the backbone atoms", which is consistent to our results. Additionally, as Fig. S11 showed that the relax step and setting pLDDT > 70 threshold can have obvious improvement on Cα atom chirality. After relax step and pLDDT threshold, the right-handed Cα rate for all amino acid except cysteine is below 0.202‰ in A501 and 0.208‰ in 3DAC, and for cysteine is below 7.016‰ in A501 and 2.037‰ in 3DAC.

For cis-peptide bonds, we compared the cis peptide bond rate to statistics from PDB structure. In our analysis in Fig. S12, the cis peptide bond rate for amide bond (include Xaa-Xaa and Pro-Xaa) is around 0.1%, while imide bond (include Xaa-Pro and Pro-Pro) is around 4%. According to previous research[7], the statistical results of PDB databased showed the cis peptide bond rate for amide bond is 0.028% and imide bond is 5.21%. Our result showed similar cis peptide bond rate propensity to the PDB database.

Discussion about the threshold of ortholog groups used in this study

In this study, we used a commonly accepted threshold to identify the ortholog group with the identity ≥ 30%, coverage ≥ 70% of bidirectional mapping, and e-value < 1e-3 as described in previous study (we cited in the manuscript)[8]. All protein pairs analyzed with functions and classified into group (i) and (ii) and (iii) are shown in Figure R2. It is obvious that the threshold for sequence cannot distinct the protein pairs with similar structures or not, which supports our statement that structure can tell more than sequence in the main text. In addition, the main statement of the metabolic modules won't be changed no matter which threshold we chose for the sequence, because our conclusions mainly depend on the similarity of structures and the sequence similarity are only use as a comparison. If we shift coverage threshold ±10-20%, it only changed the classification of

a few protein pairs within group (i) and (ii) (**Supplementary Figure 13**). For our statement, both group (i) and (ii) are conserved and most likely from the common ancestor of archaea and bacteria, and the group (ii) is more variable in sequence than group (i). So, the main conclusion won't be changed. For the sequence identity, the current threshold of 30% identity is the commonly used to identity orthologs, and it is believed to be associated with the evolution time: the change from 30% to 20% might correspond to a billion-year divergence time change[9]. Even if we shift the identity threshold ±10-20%, the different metabolic modules can also be identified based on the structure similarity.

Supplementary Figures

Supplementary Figure 1. (A) Metabolic capabilities on carbon and energy sources tested in A501 and 3DAC. The tested media of carbon sources are based on TRM basal medium with all 20 amino acids (denoted as “20aa”). “20aa-X” represents the media with removal of individual amino acids, and X represents one of the 20 amino acids. “20aa+Y” represents the media with extra carbon source supplied with 20 amino acids, and Y represents D-glucose and maltose. The tested media of energy sources are based on TRM medium without sulfur (denoted as “TRM-S”). “TRM+Z” represents the media with extra energy source supplied, and Z represents elemental sulfur (S) and L-cysteine (Cys). “+” in light color represents similar growth yields compared with the 20aa condition; “++” in dark color represents at least 1.5-fold higher growth yields than 20aa; “-” in blank represents no growth. Groups numbered with (i) to (iii) are corresponding to the groups in main text.

	**Source**	**3DAC**	**A501**
Carbon	20aa	+	+
	20aa-Ala	+	+
	20aa-Gln	+	+
	20aa-Glu	+	+
	20aa-Ser	+	+
	20aa-Gly	+	+
	20aa-Ile	+	+
	20aa-Val	+	+
	20aa-Asn	+	+
	20aa-Leu	-	+
	20aa-Asp	+	-
	20aa-Lys	-	+
	20aa-Pro	-	+
	20aa-Cys	-	+
	20aa-Arg	-	-
	20aa-His	-	-
	20aa-Met	-	-
	20aa-Phe	-	-
	20aa-Tyr	-	-
	20aa-Trp	-	-
	20aa-Thr	-	-
	20aa+Glucose	++	++
	20aa+Maltose	+	++
	Yeast Extract	++	++
	Typtone	++	++
Energy	TRM - S	+	+
	TRM +S	++	++

Supplementary Figure 2. Proteome-wide structure predictions of A501 and 3DAC. **(A)** Composition protein structure predictions with average pLDDT per protein in A501 and 3DAC, compared with the model organisms. **(B-C)** pLDDT score present high consistency in fraction and high correlation when using different models (compare model 3 to all other models) in AlphaFold2-based pipeline **(D)**.

(A) pLDDT per protein in A501 and 3DAC results (model 3) and other species from AlphaFold database

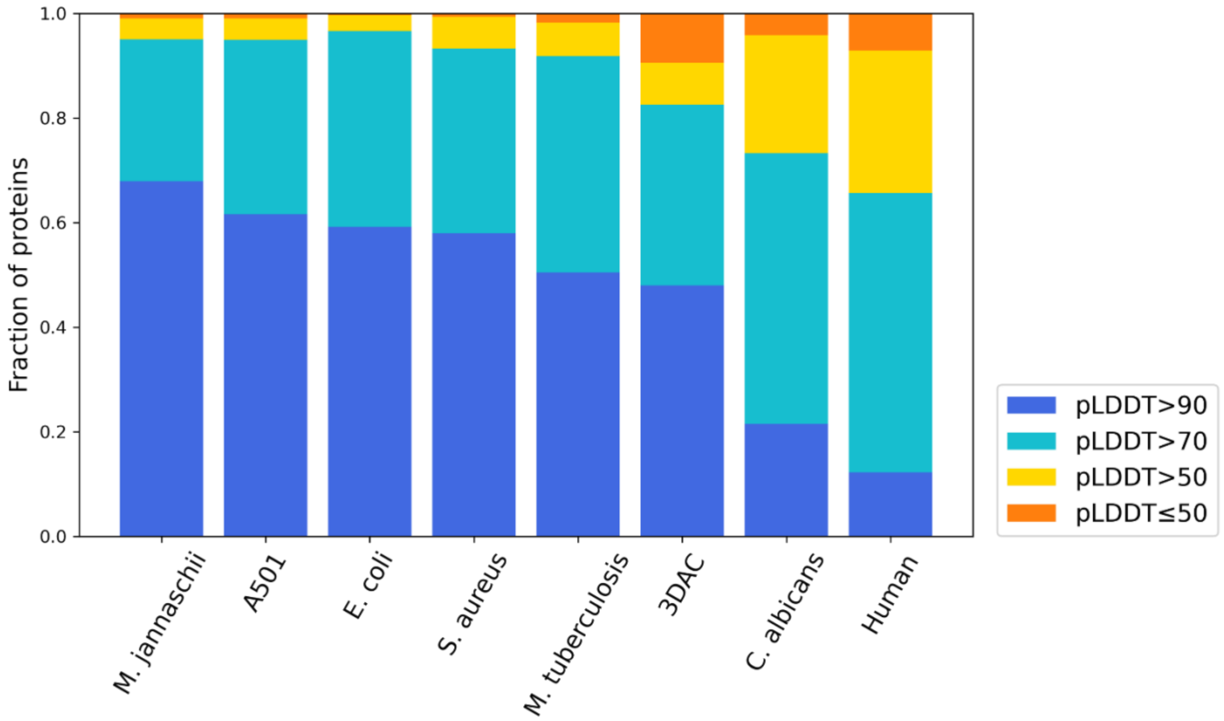

(B) pLDDT per protein of different AlphaFold models in A501 and 3DAC results

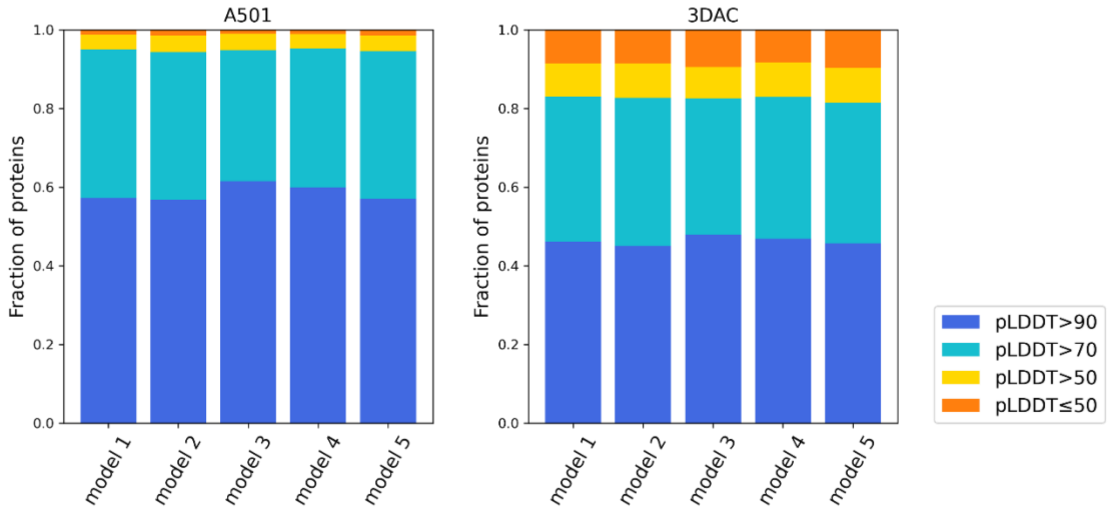

(C) pLDDT per residue of different AlphaFold models in A501 and 3DAC results

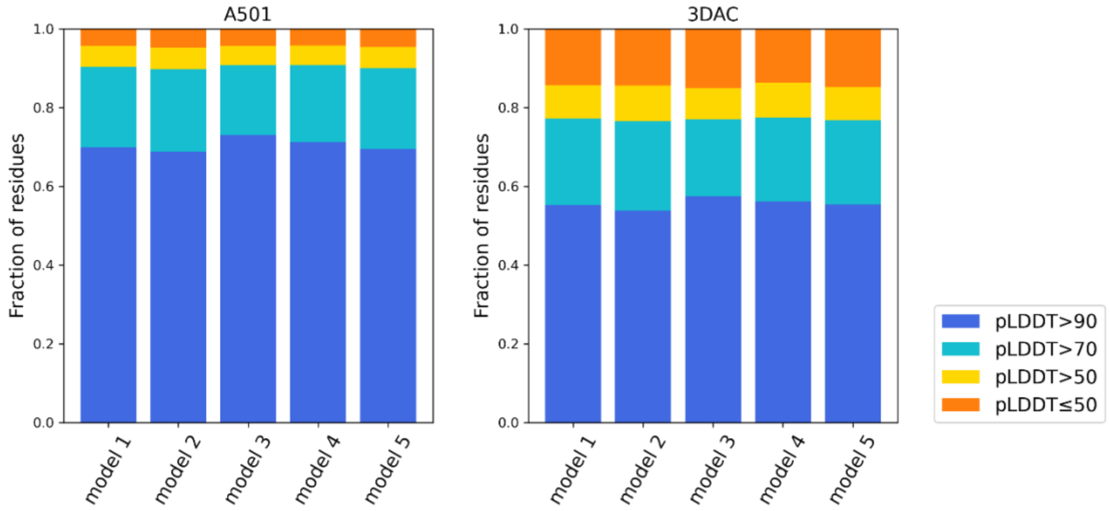

(D) Correlation of average protein pLDDT of different AlphaFold models

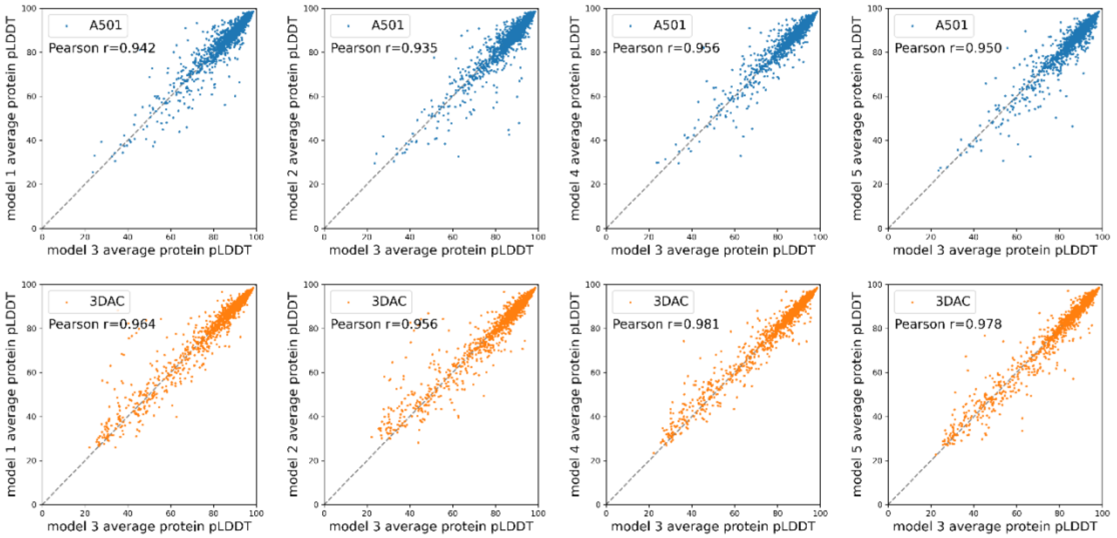

Supplementary Figure 3. Statistical comparison of the number of hydrogen bonds between each protein pairs in A501 and 3DAC in different function categories. Function categories are based on the COG categories and curated by further annotations.

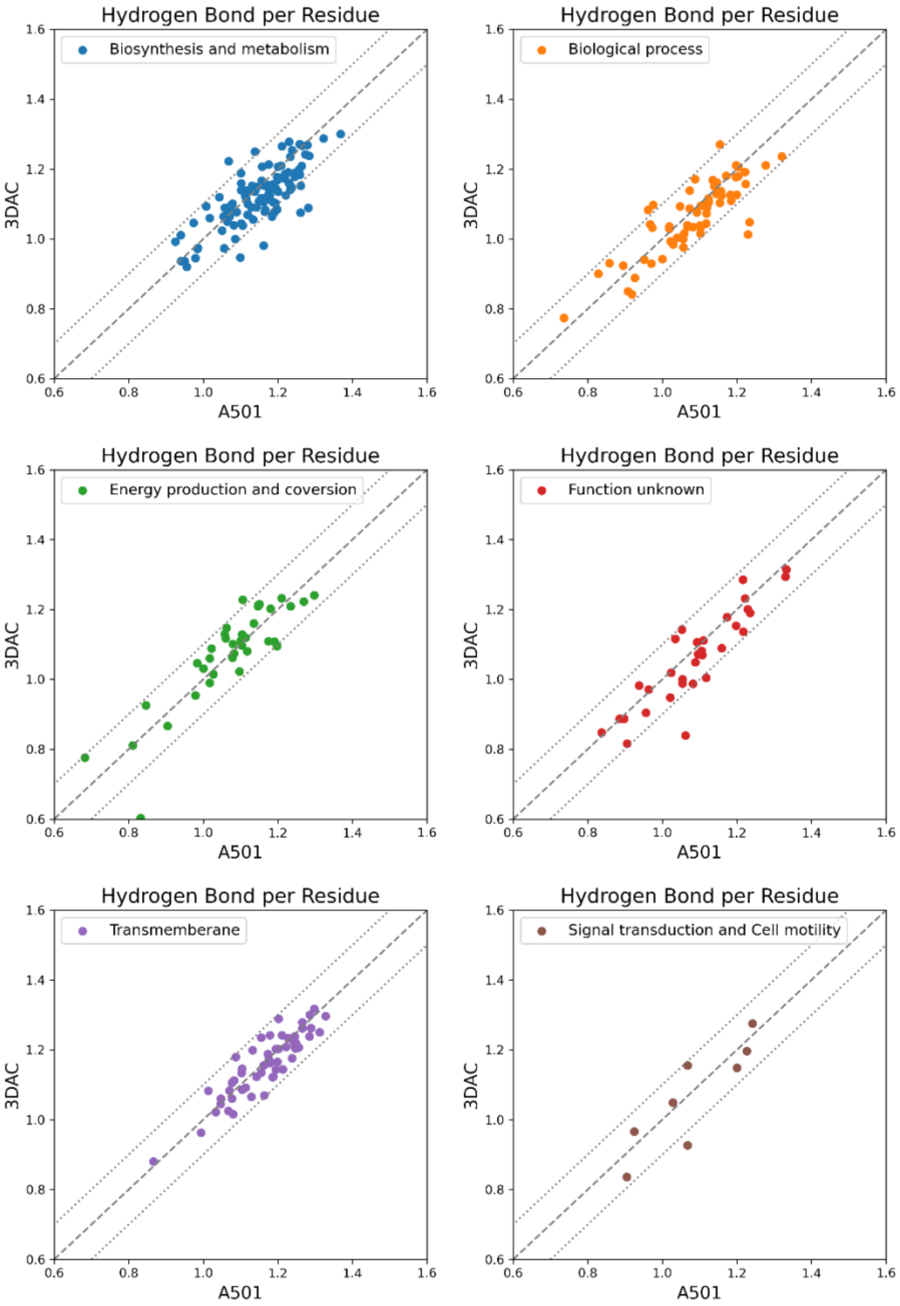

Supplementary Figure 4. Population of each type of amino acid in the proteins in A501 (blue) and 3DAC (red).

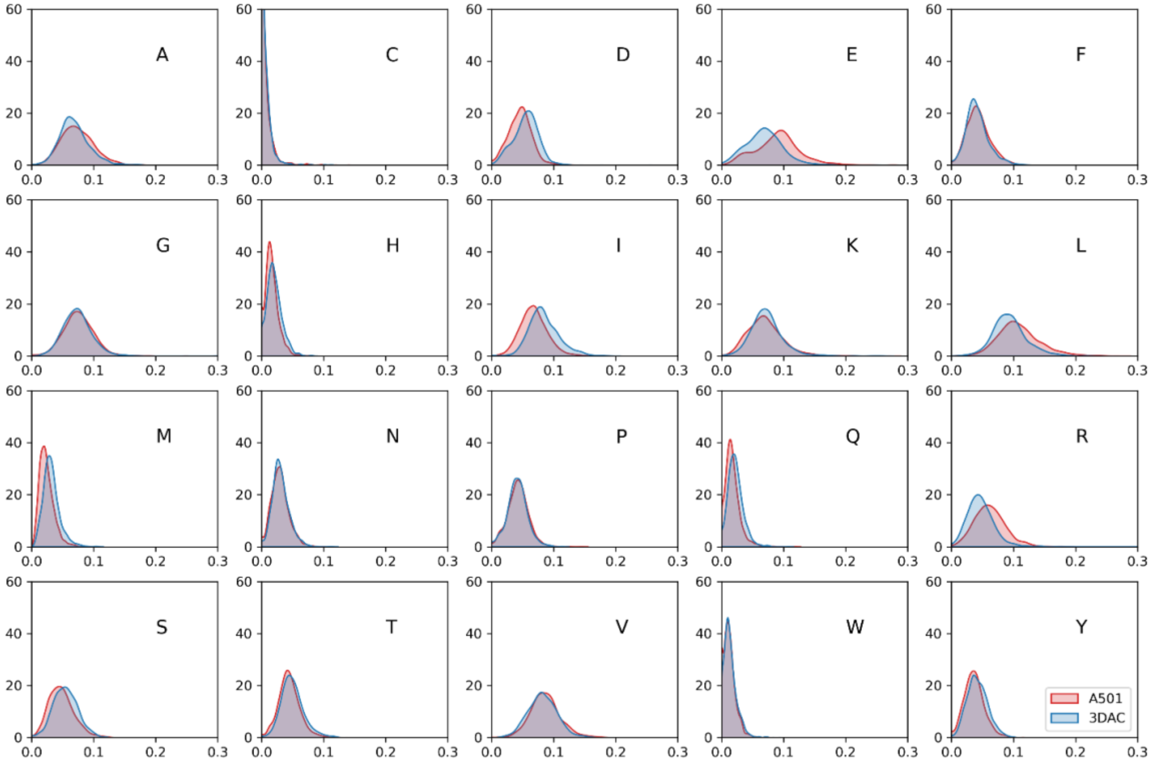

Supplementary Figure 5. Structure prediction of protein pairs with the same function but different structures (normalized RMSD > 6 Å) between A501 and 3DAC. The A501 protein structures are colored in red, while 3DAC structures are represented in blue. The references shown in green, are the structures from protein data bank (pdb), which have the lowest RMSD divergence with the target structure.

(A) Lysyl-tRNA synthetase (EC 6.1.1.6). Representative structures for A501 protein (red) is 1irx (*Pyrococcus horikoshii*, green on top), while for 3DAC protein (blue) is ibbu (*E. coli*, green on bottom).

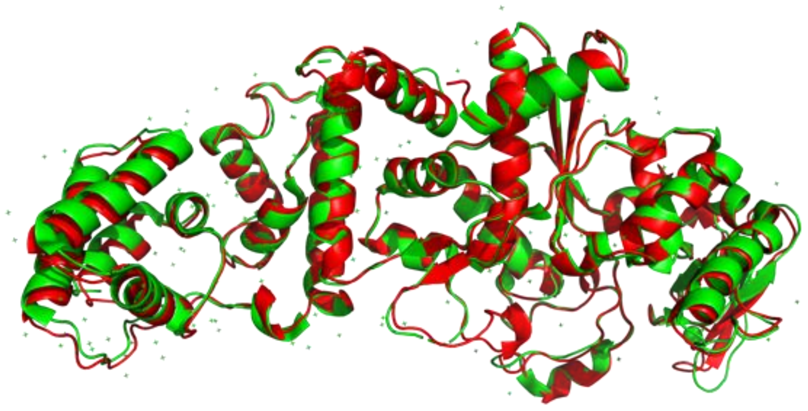

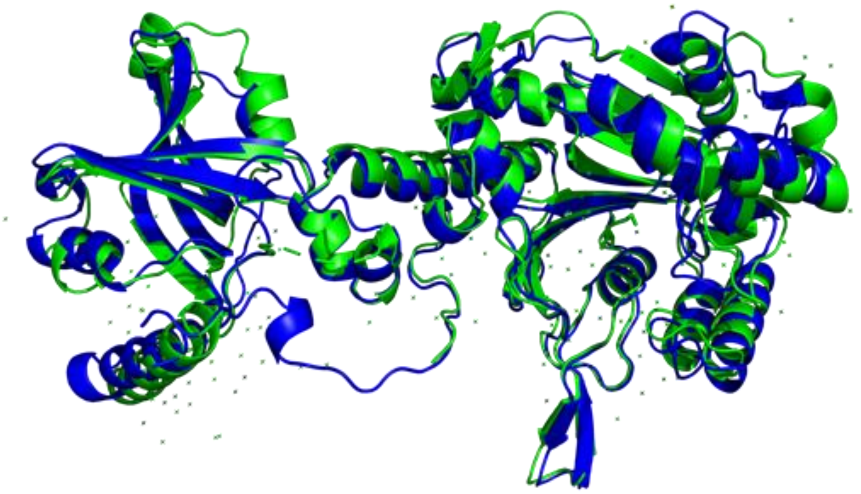

(B) Glycyl-tRNA synthetase (EC 6.1.1.14). Representative structures for A501 protein (red) is 4qej (*Homo sapiens*, green on top), while for 3DAC protein (blue) is 1j5w (*Thermotoga maritima*, green on bottom).

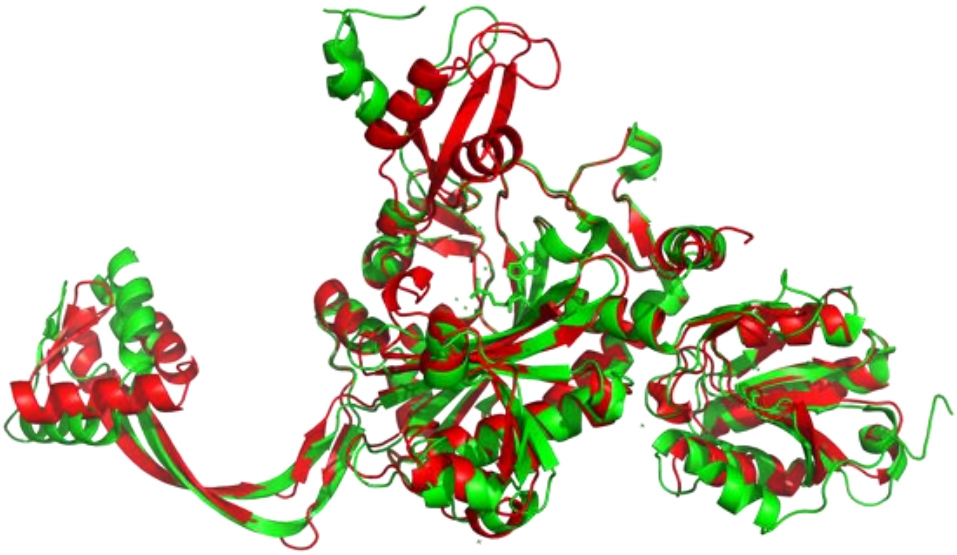

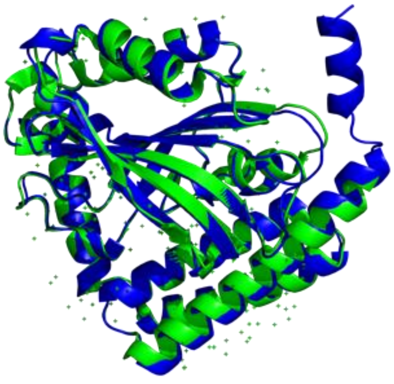

(C) Glucose-6-phosphate isomerase (EC 5.3.1.9). Representative structures for A501 protein (red) is 2gc1 (*Pyrococcus furiosus*, green on top), while for 3DAC protein (blue) is 1hox (*Oryctolagus cuniculus*, green on bottom).

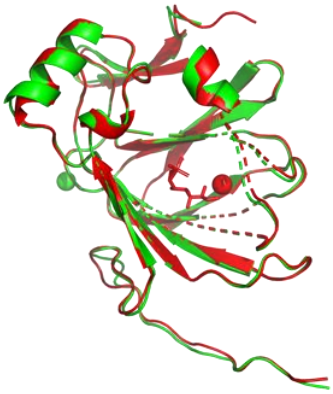

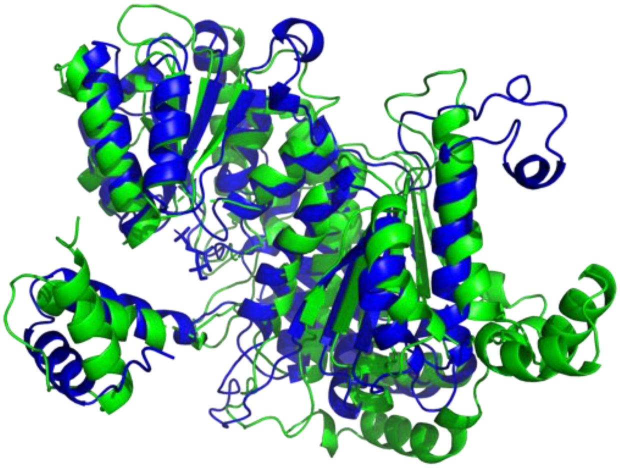

(D) Inosinate cyclohydrolase (EC 3.5.4.10). Representative structures for A501 protein (red) is 2NTK (*Methanothermobacter thermautotrophicus*, green on top), while for 3DAC protein (blue) is 1ZCZ (*Thermotoga maritima*, green on bottom).

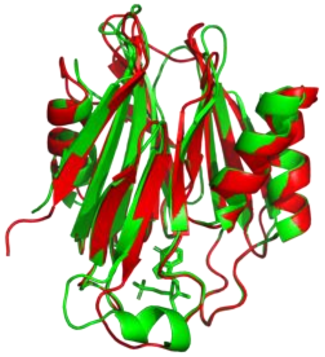

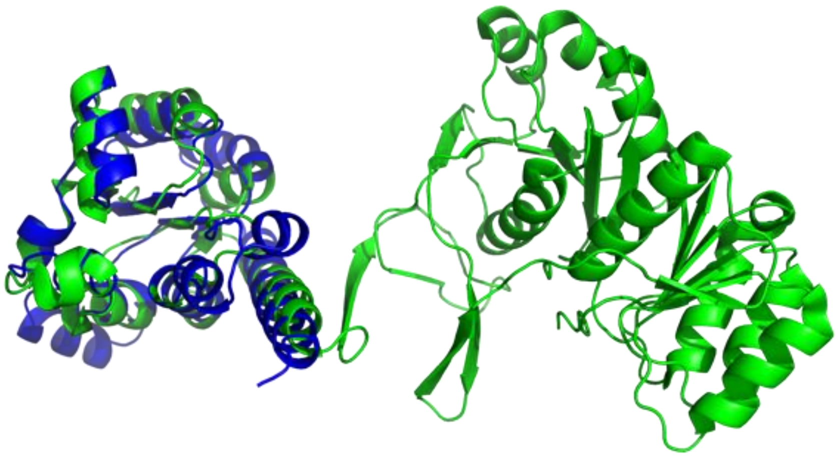

(E) Glycinamide ribonucleotide transformylase (EC 2.1.2.2). Representative structures for A501 protein (red) is 2QK4 (*Homo sapiens*, green on top), while for 3DAC protein (blue) is 1MEN (*Homo sapiens*, green on bottom).

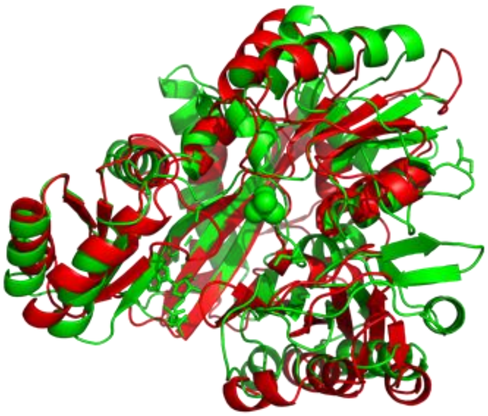

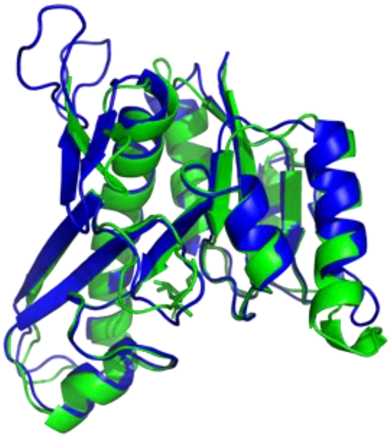

(F) Methionyl-tRNA synthetase (EC 6.1.1.10). Representative structures for A501 protein (red) is 1rqg (*Pyrococcus abyssi*, green on top), while for 3DAC protein (blue) is 1qqt (*E. coli*, green on bottom).

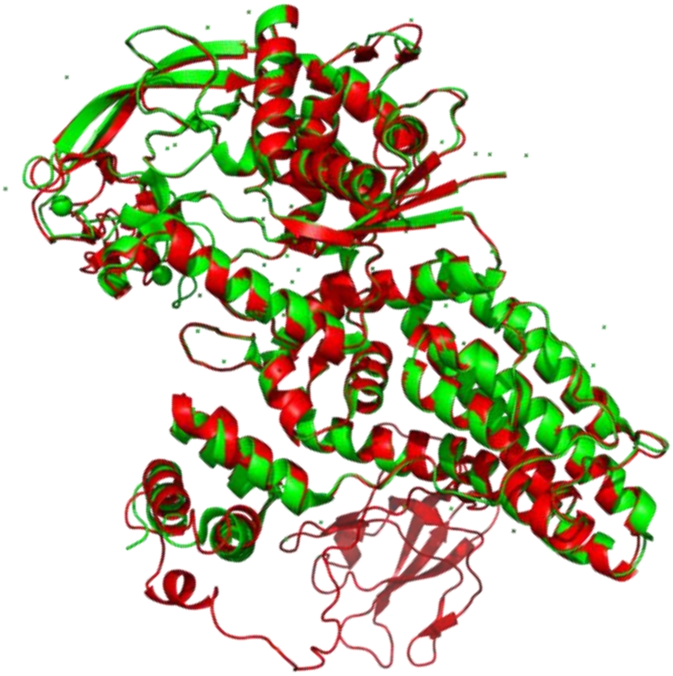

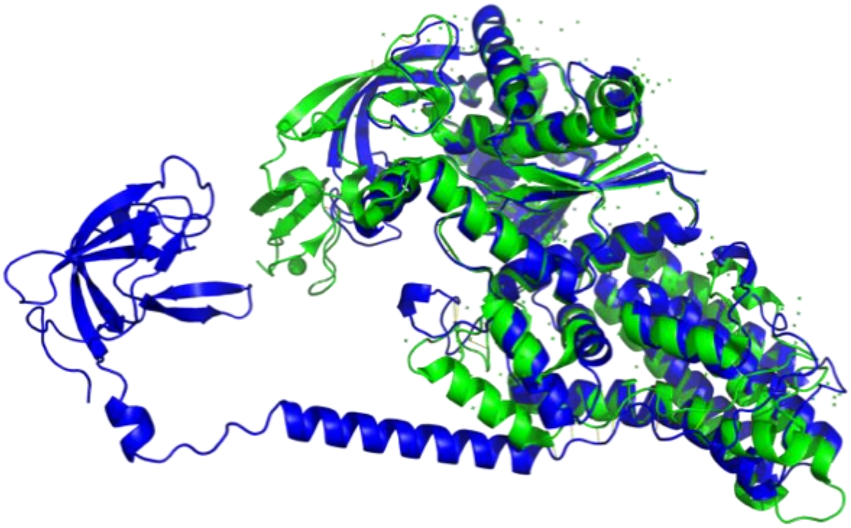

(G) Ribose-5-phosphate isomerase (EC 5.3.1.6). Representative structures for A501 protein (red) is 1o8b (*E. coli*, green on top), while for 3DAC protein (blue) is 2vvp (*Mycobacterium tuberculosis*, green on bottom).

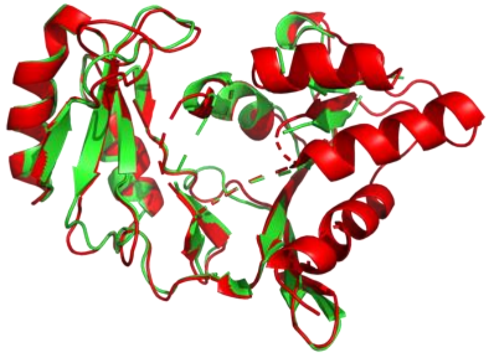

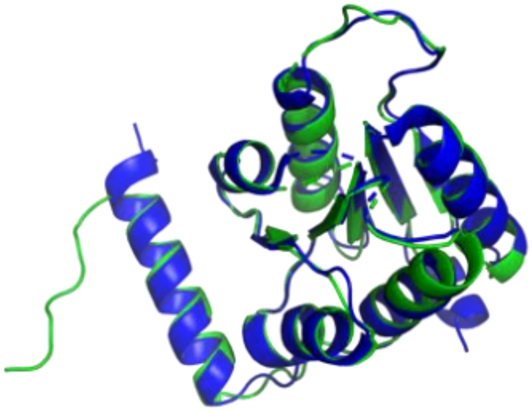

(H) Leucyl-tRNA synthetase (EC 6.1.1.4). Representative structures for A501 protein (red) is 1wkb (*Pyrococcus horikoshii*, green on top), while for 3DAC protein (blue) is 1h3n (*Thermus thermophilus*, green on bottom).

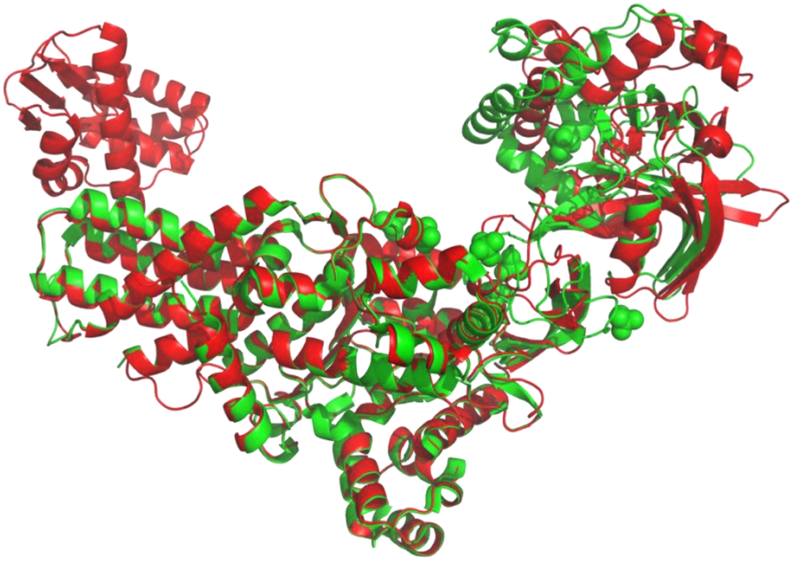

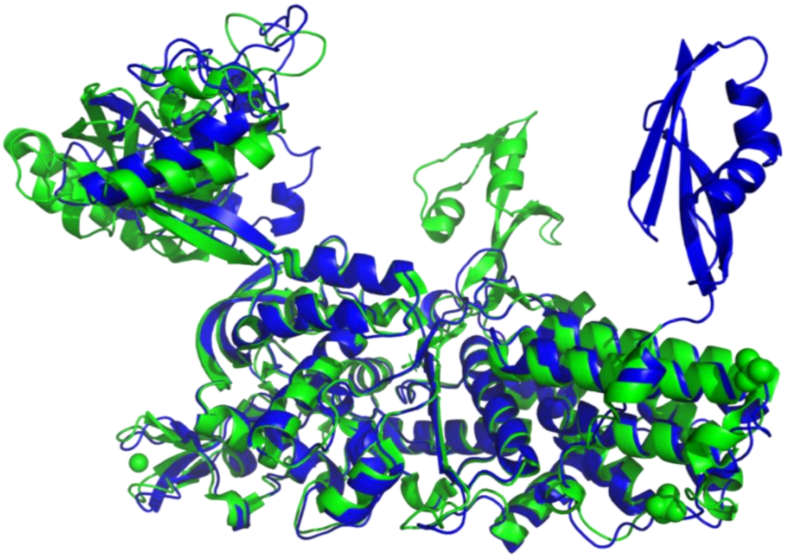

(I) Phenylalanyl-tRNA synthetase (EC 6.1.1.20). Representative structures for A501 protein (red) is 1b70 (*Thermus thermophilus*, green on top), while for 3DAC protein (blue) is 4p71 (*Pseudomonas aeruginosa*, green on bottom).

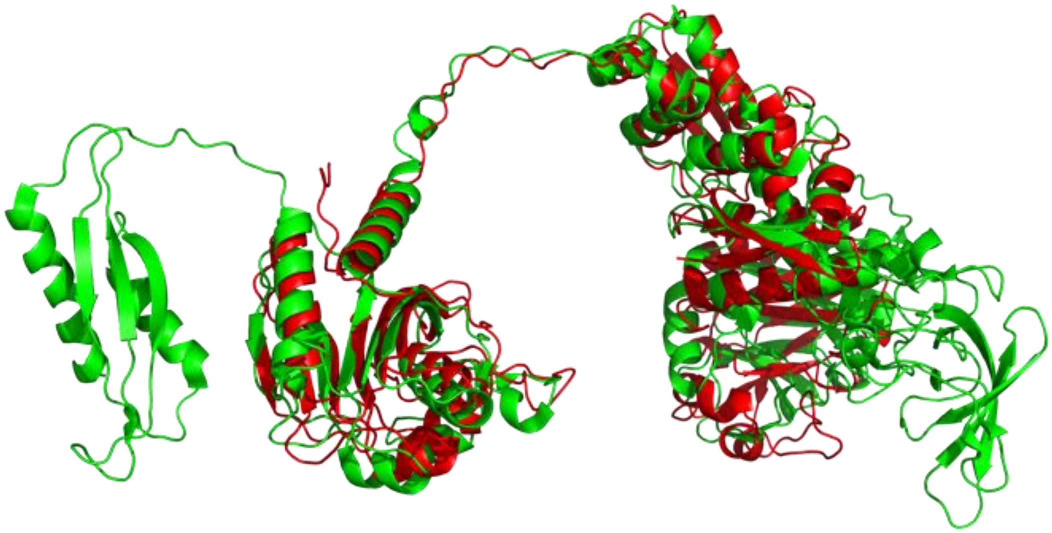

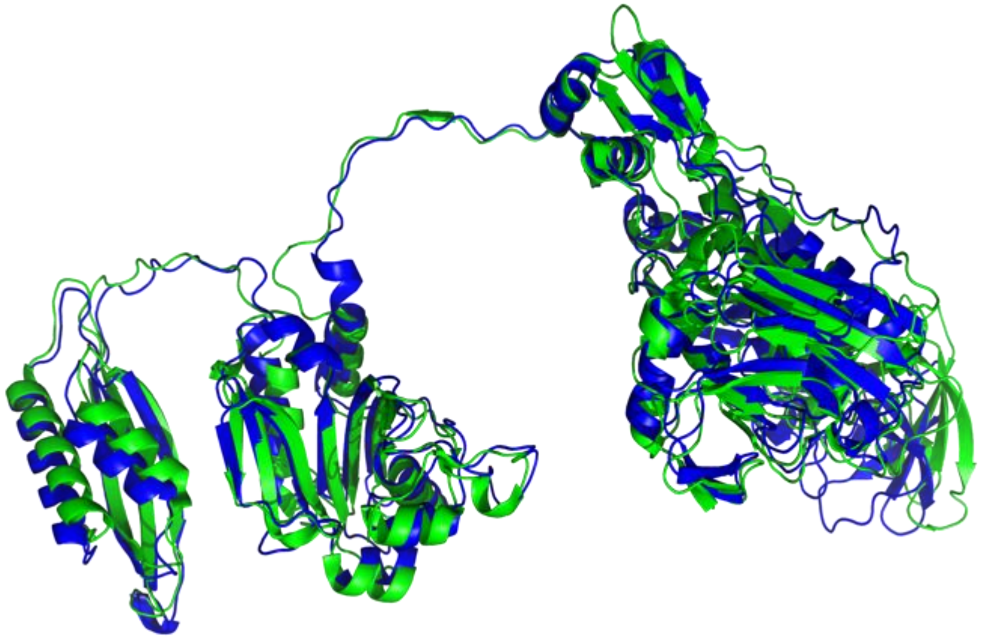

(J) Fructose-bisphosphate aldolase (EC 4.1.2.13). Representative structures for A501 protein (red) is 1w8s (*Thermoproteus tenax*, green in left), while for 3DAC protein (blue) is 1b57 (*E. coli*, green in right).

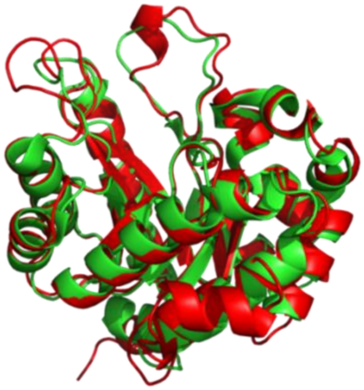

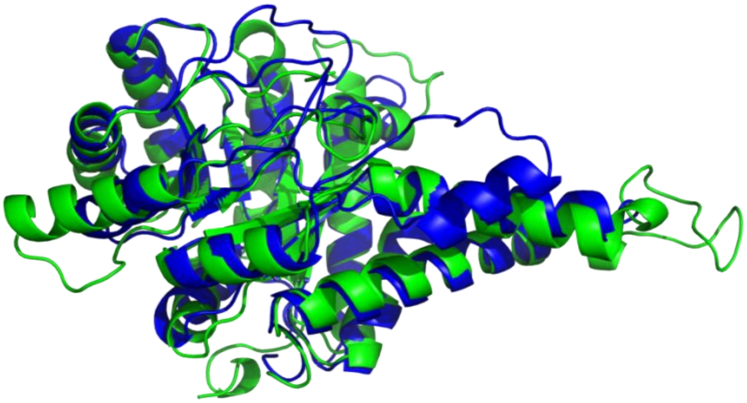

Supplementary Figure 6. Phylogenetic analysis of seven key enzymes in glycolysis/glycogenesis pathway among A501, 3DAC, other 12 archaeal and 12 bacterial strains. Phylogenetic tree was built using IQ-Tree v1.6.6 with the model LG+C60+F+G with a bootstrap value of 1000, and the bootstrap larger than 0.8 were shown as a grey square on the tree branches. Archaeal genes are represented in dark pink branches, while bacterial genes are represented in black. The seven enzymes serve as outgroups for each other. Followings present each enzyme one by one.

(A) Glyceraldehyde-3-phosphate dehydrogenase (EC 1.2.1.59/ 1.2.1.12).

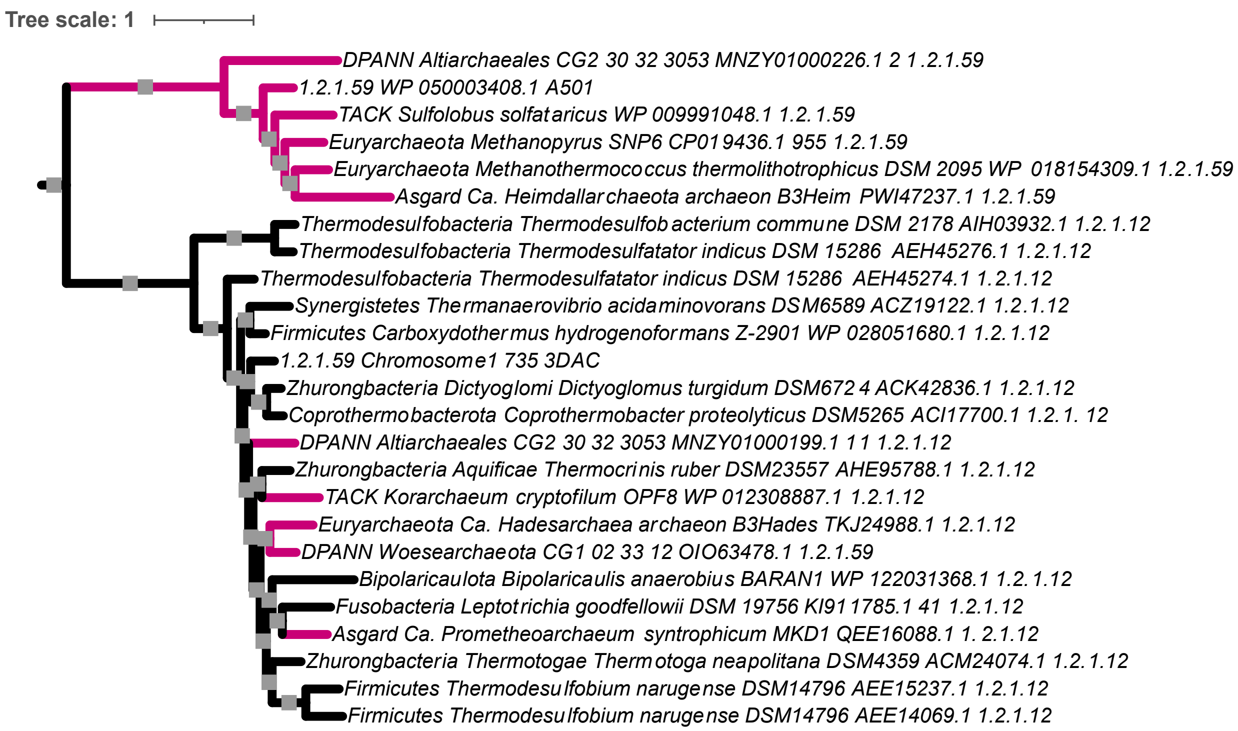

(B) Triose-phosphate isomerase (EC 5.3.1.1).

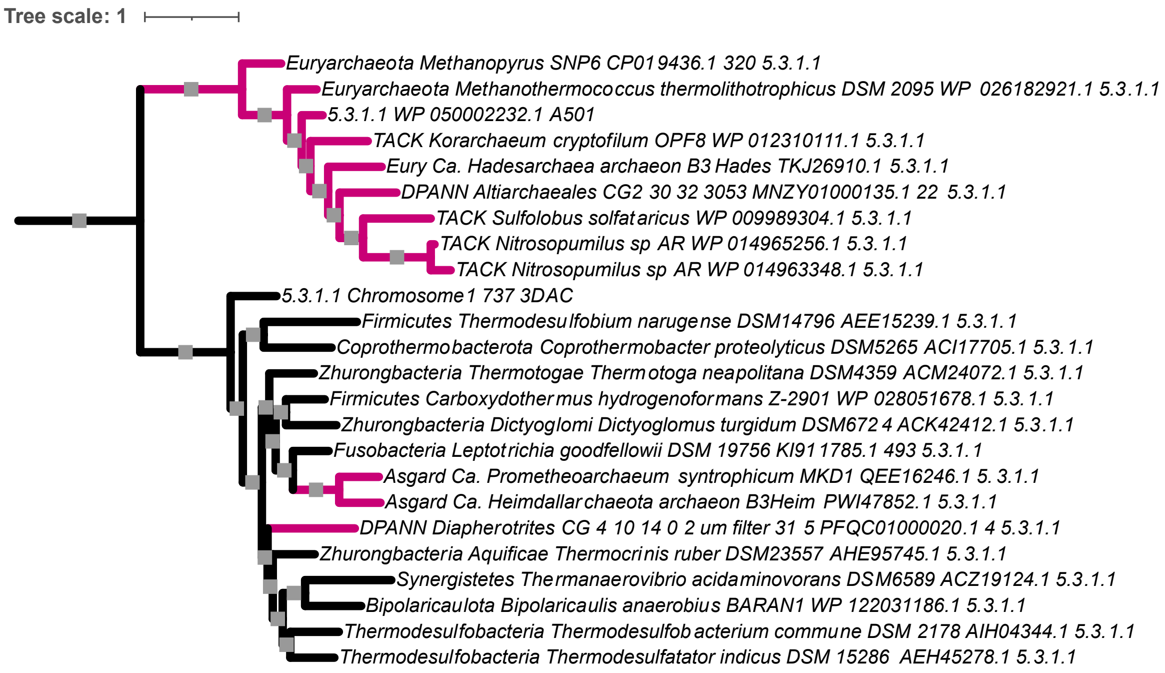

(C) Phosphoglycerate kinase (EC 2.7.2.3).

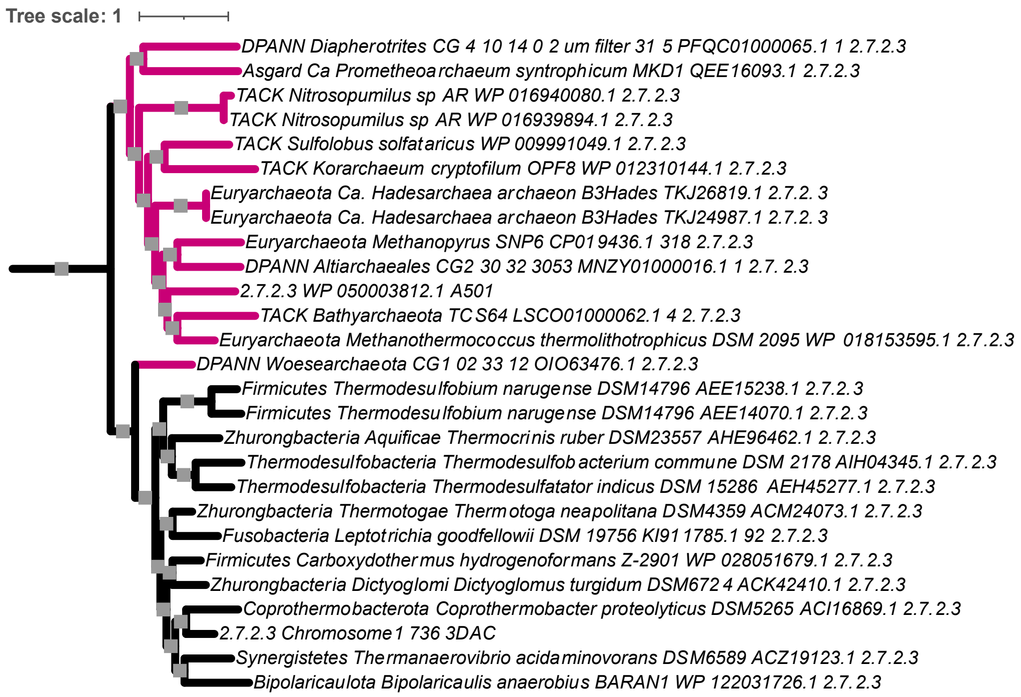

(D) Phosphoglycerate phosphomutase (EC 5.4.2.12).

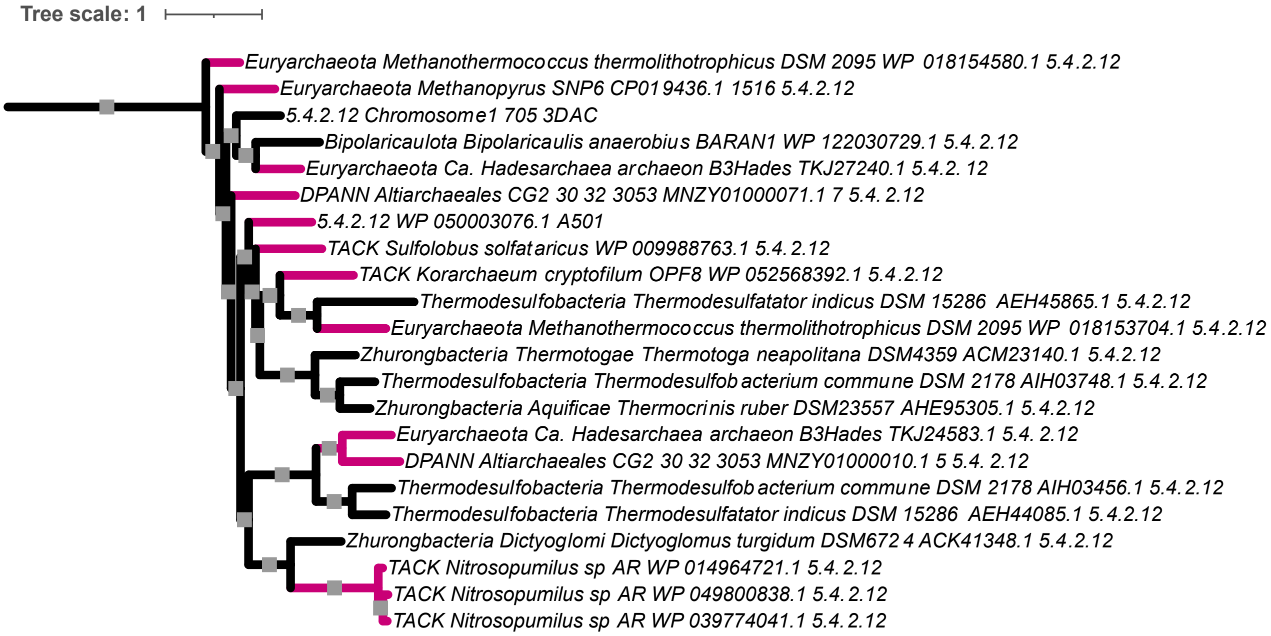

(E) Enolase (EC 4.2.1.11).

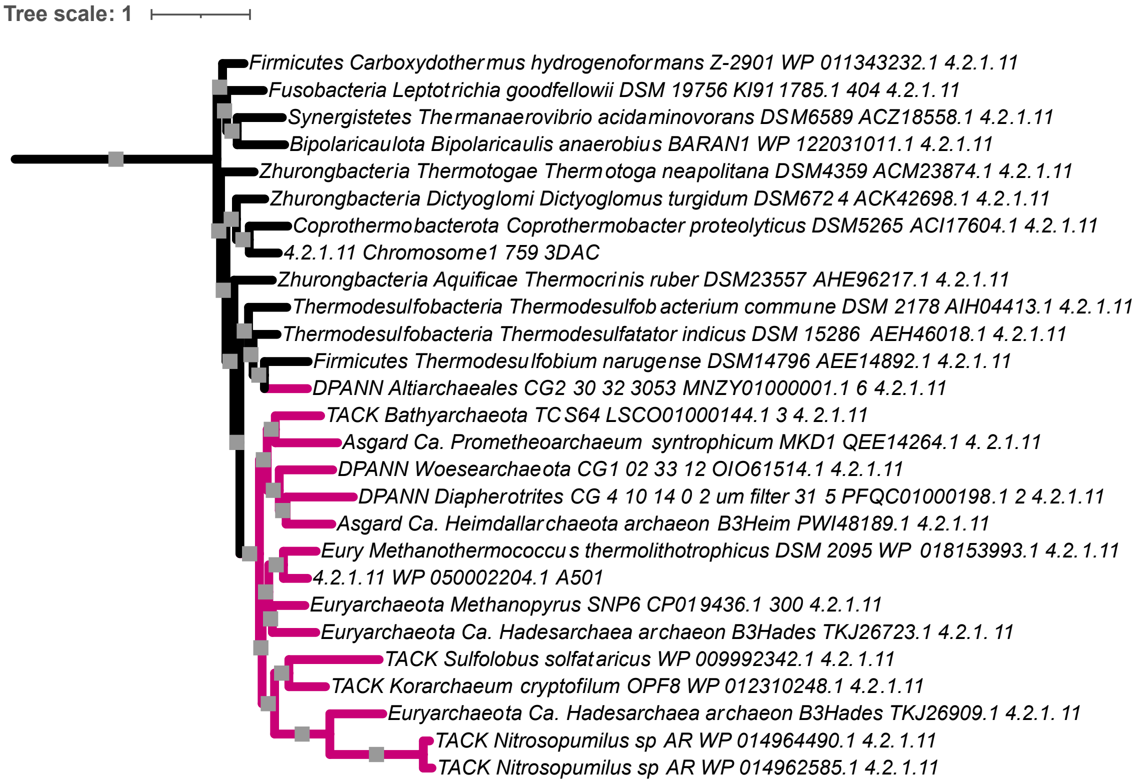

(F) Pyruvate kinase (EC 2.7.1.40).

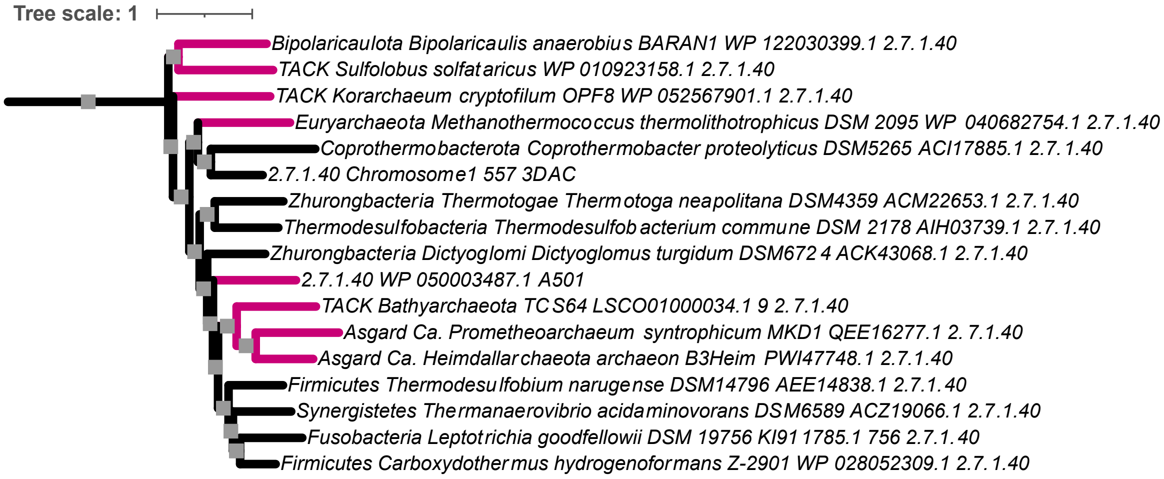

(G) 2-oxoacid oxidoreductase (EC 1.2.7.11).

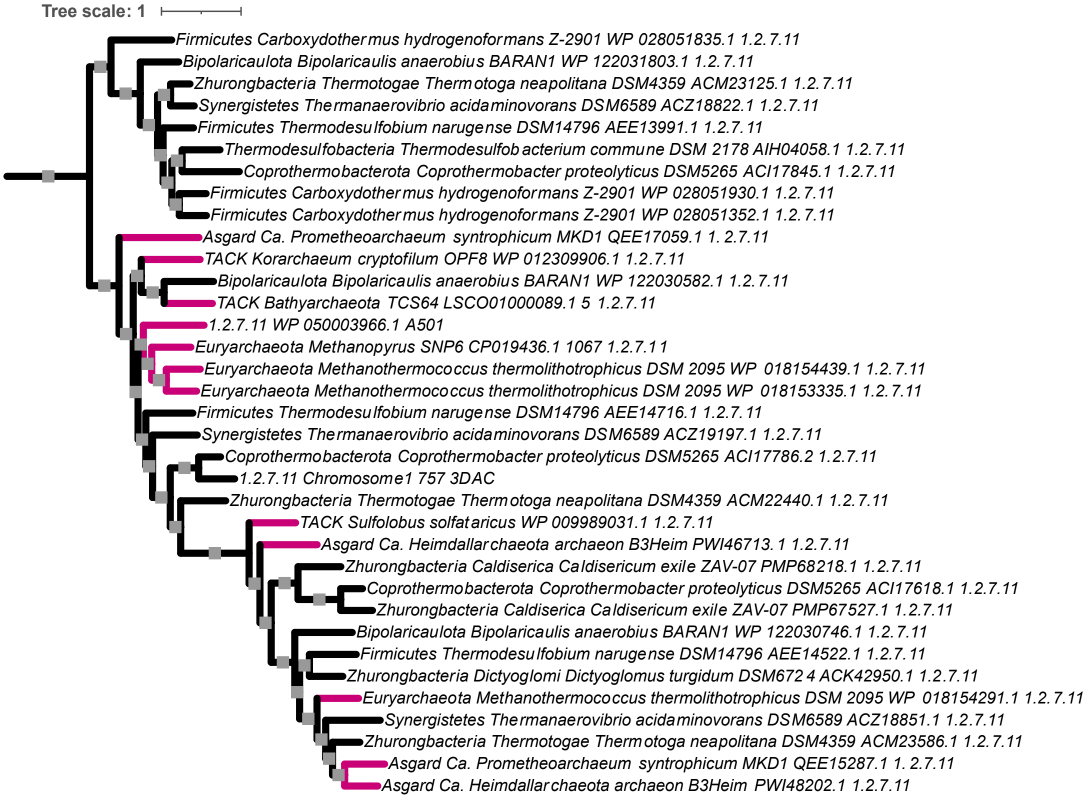

Supplementary Figure 7. Statistical analysis of similarity and difference of structures and sequences among seven key enzymes in glycolysis/glycogenesis pathway among A501, 3DAC, other 12 archaeal and 12 bacterial strains. Followings present the heatmap of structure similarities and sequence similarities one by one.

(A) Glyceraldehyde-3-phosphate dehydrogenase (EC 1.2.1.59/ 1.2.1.12): structure

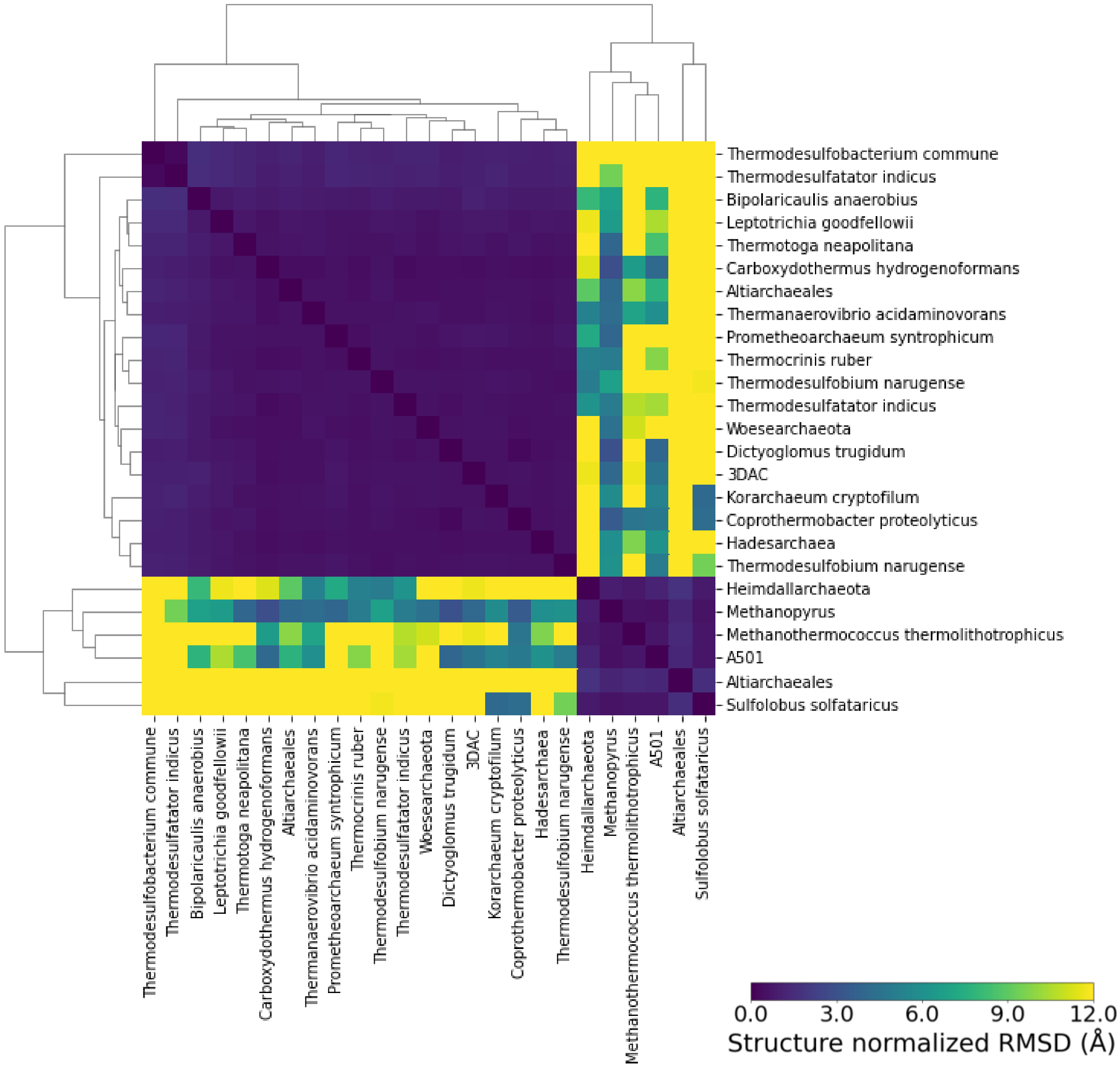

(A) Glyceraldehyde-3-phosphate dehydrogenase (EC 1.2.1.59/ 1.2.1.12): sequence

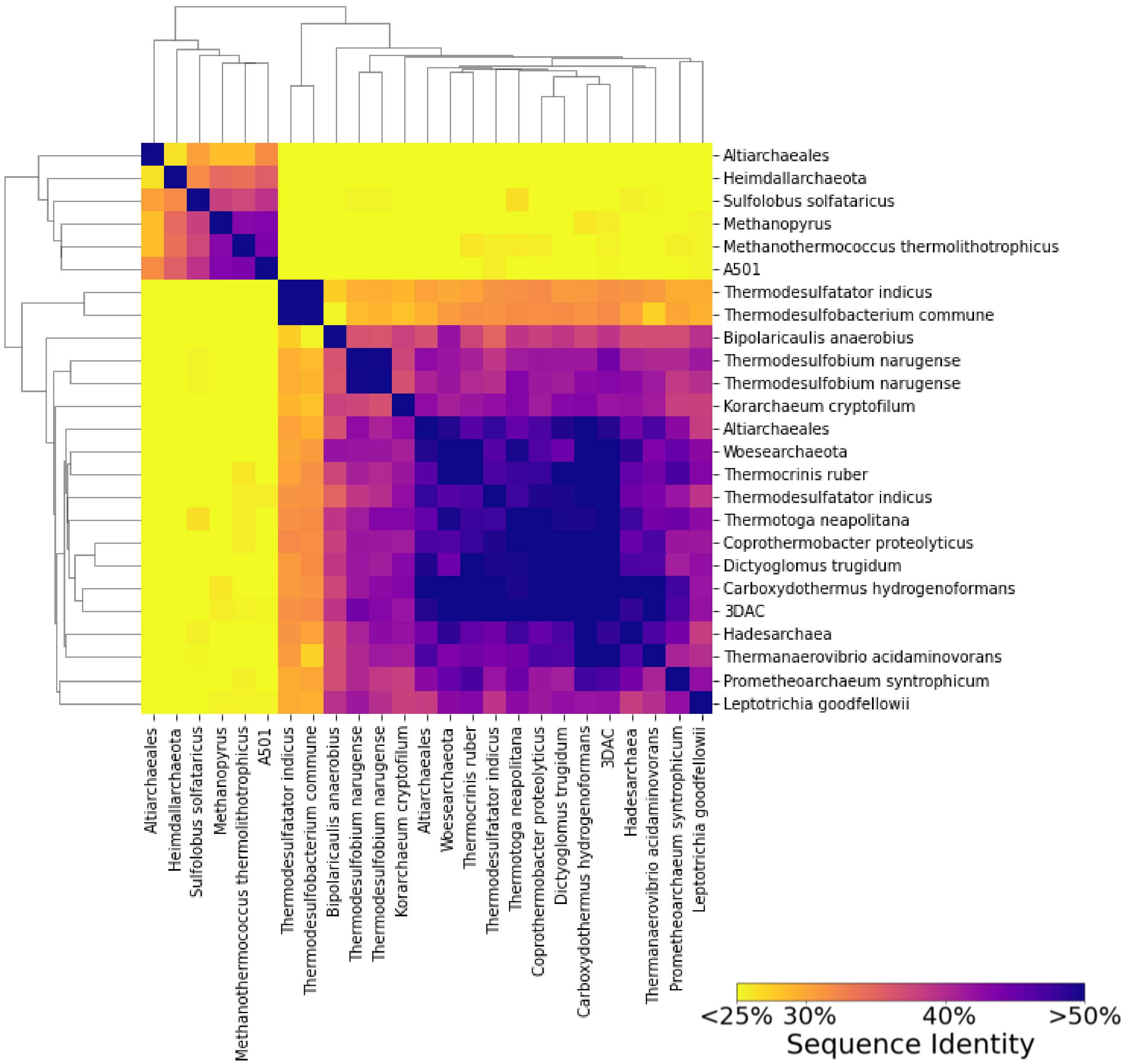

(B) Triose-phosphate isomerase (EC 5.3.1.1): structure

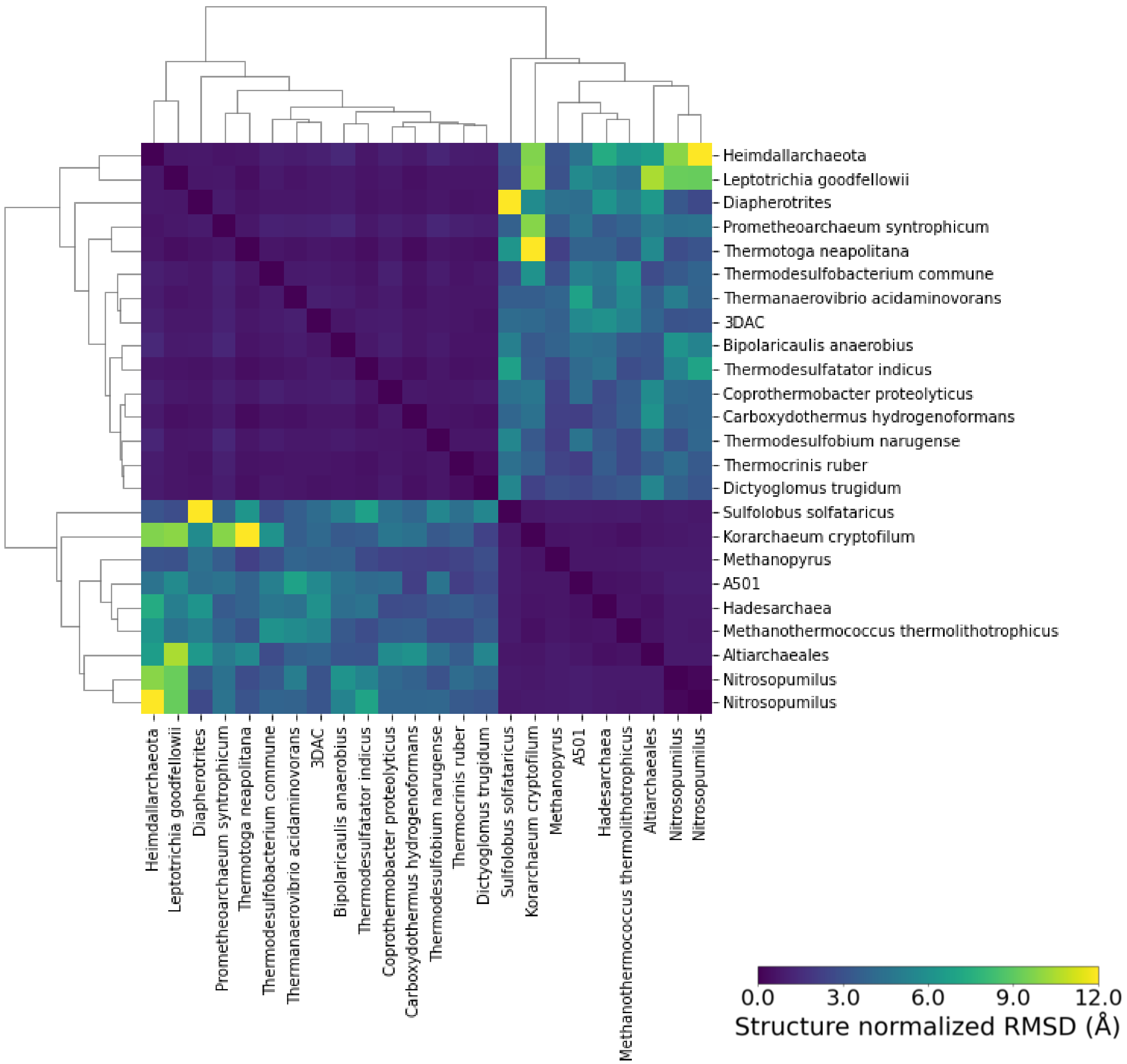

(B) Triose-phosphate isomerase (EC 5.3.1.1): sequence

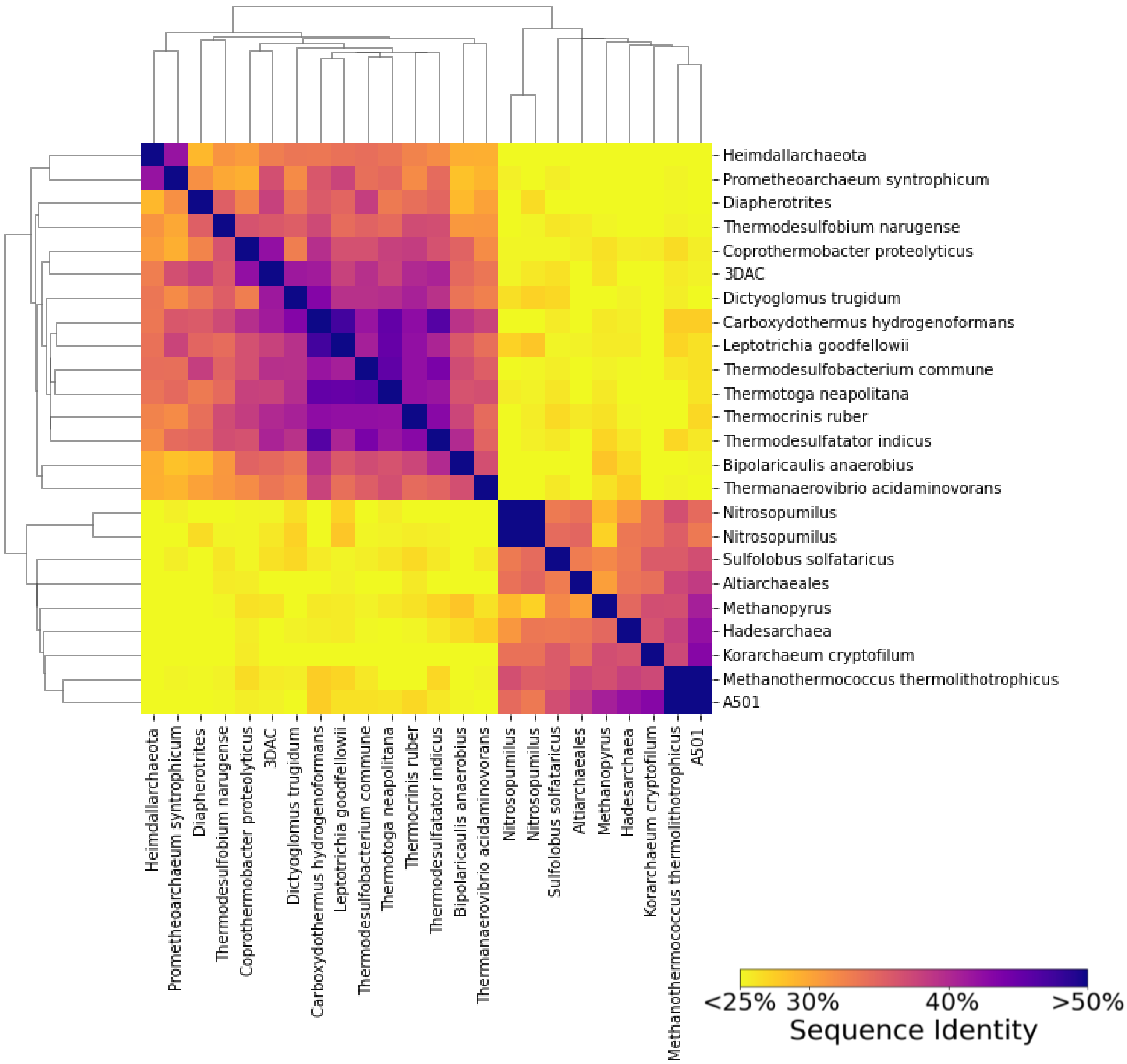

(C) Phosphoglycerate kinase (EC 2.7.2.3): structure

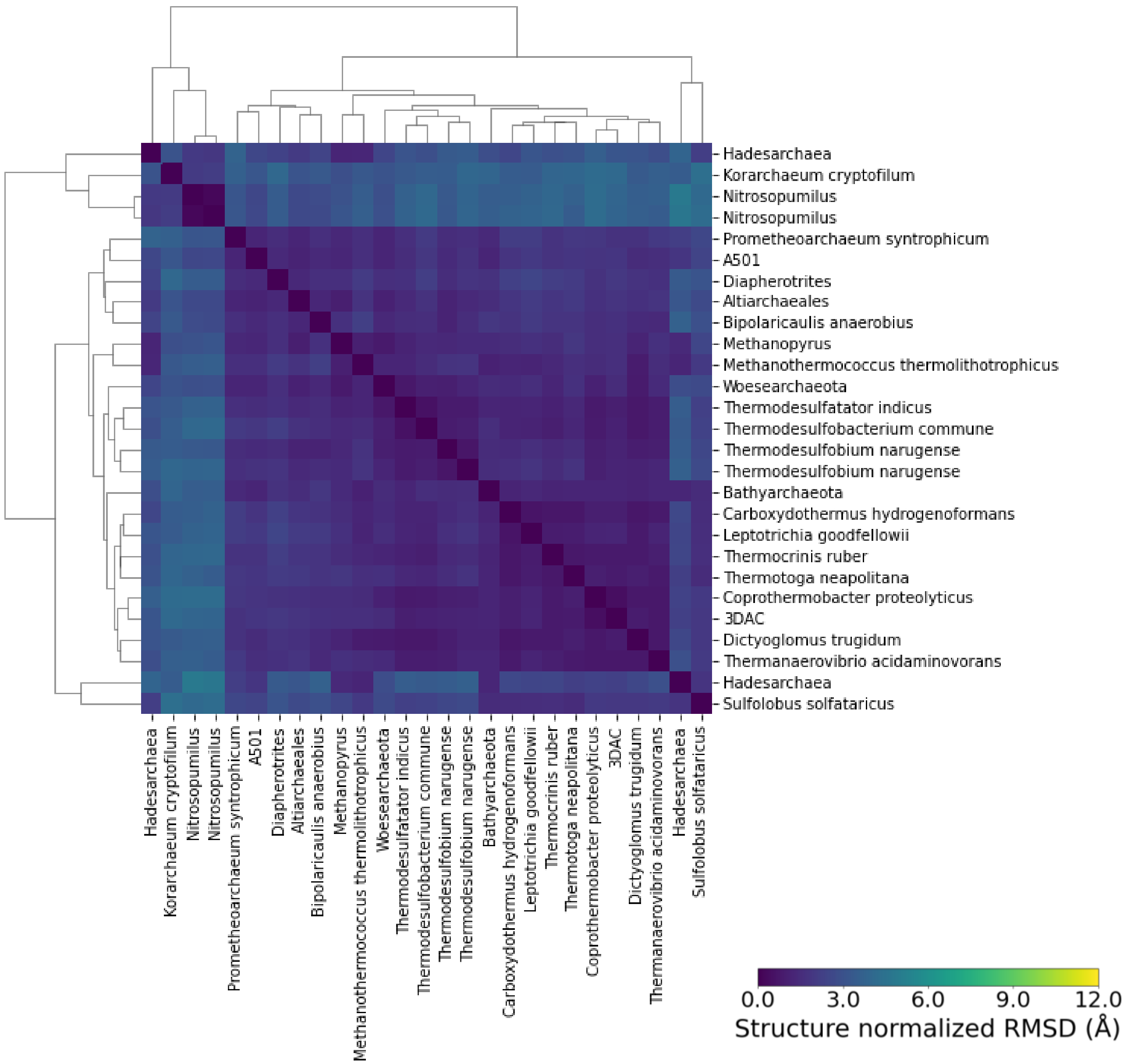

(C) Phosphoglycerate kinase (EC 2.7.2.3): sequence

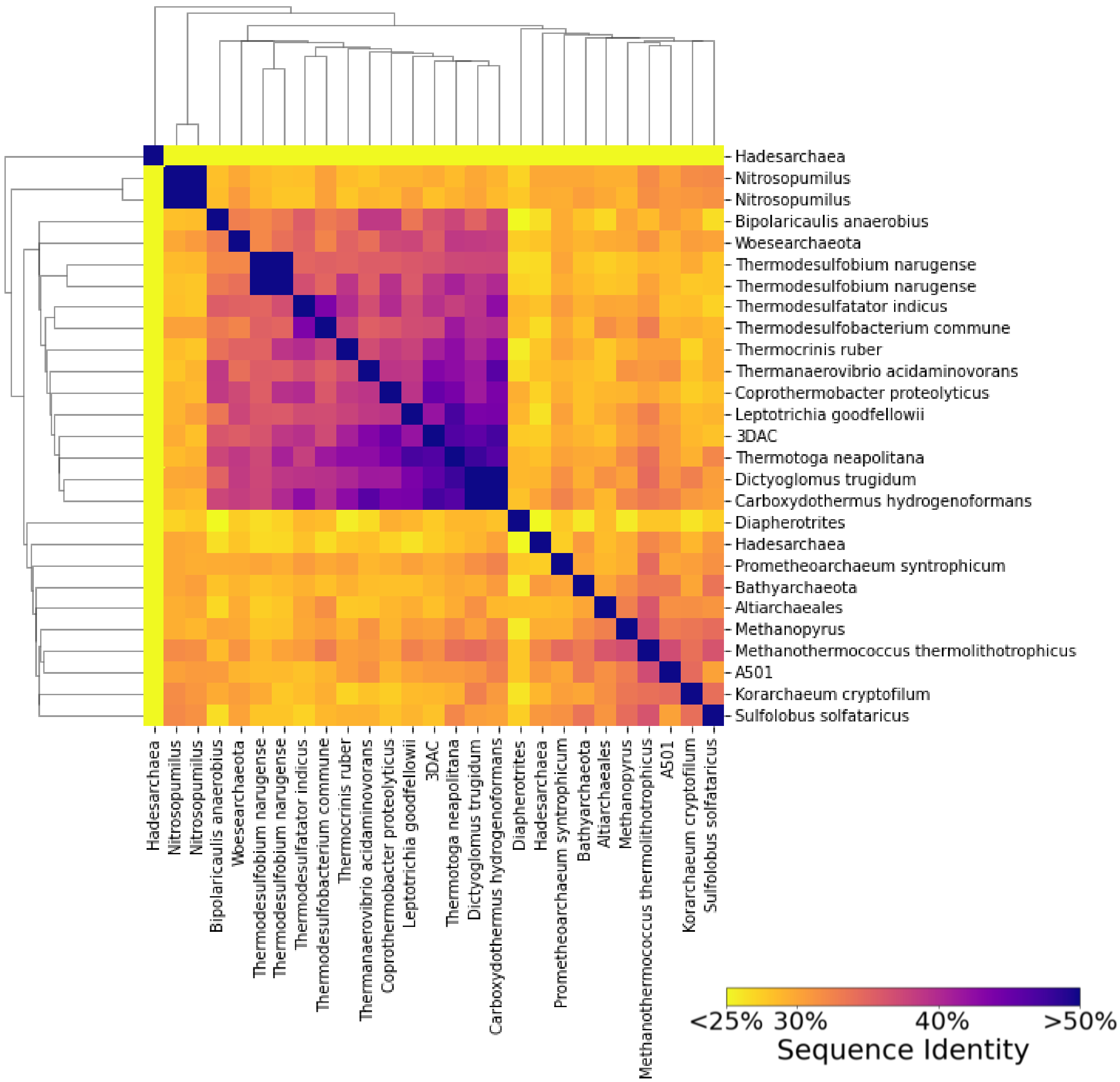

(D) Phosphoglycerate phosphomutase (EC 5.4.2.12): structure

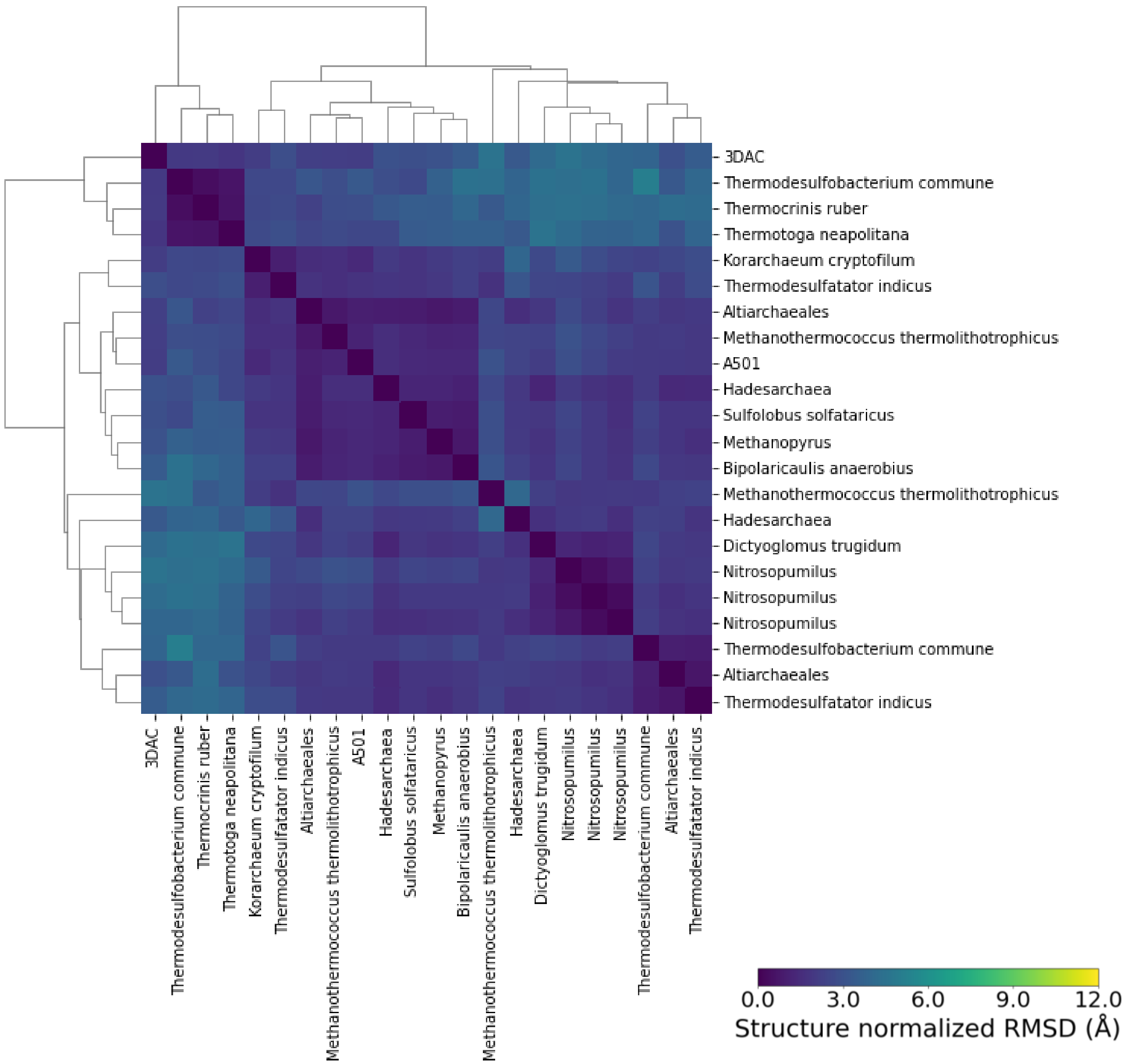

(D) Phosphoglycerate phosphomutase (EC 5.4.2.12): sequence

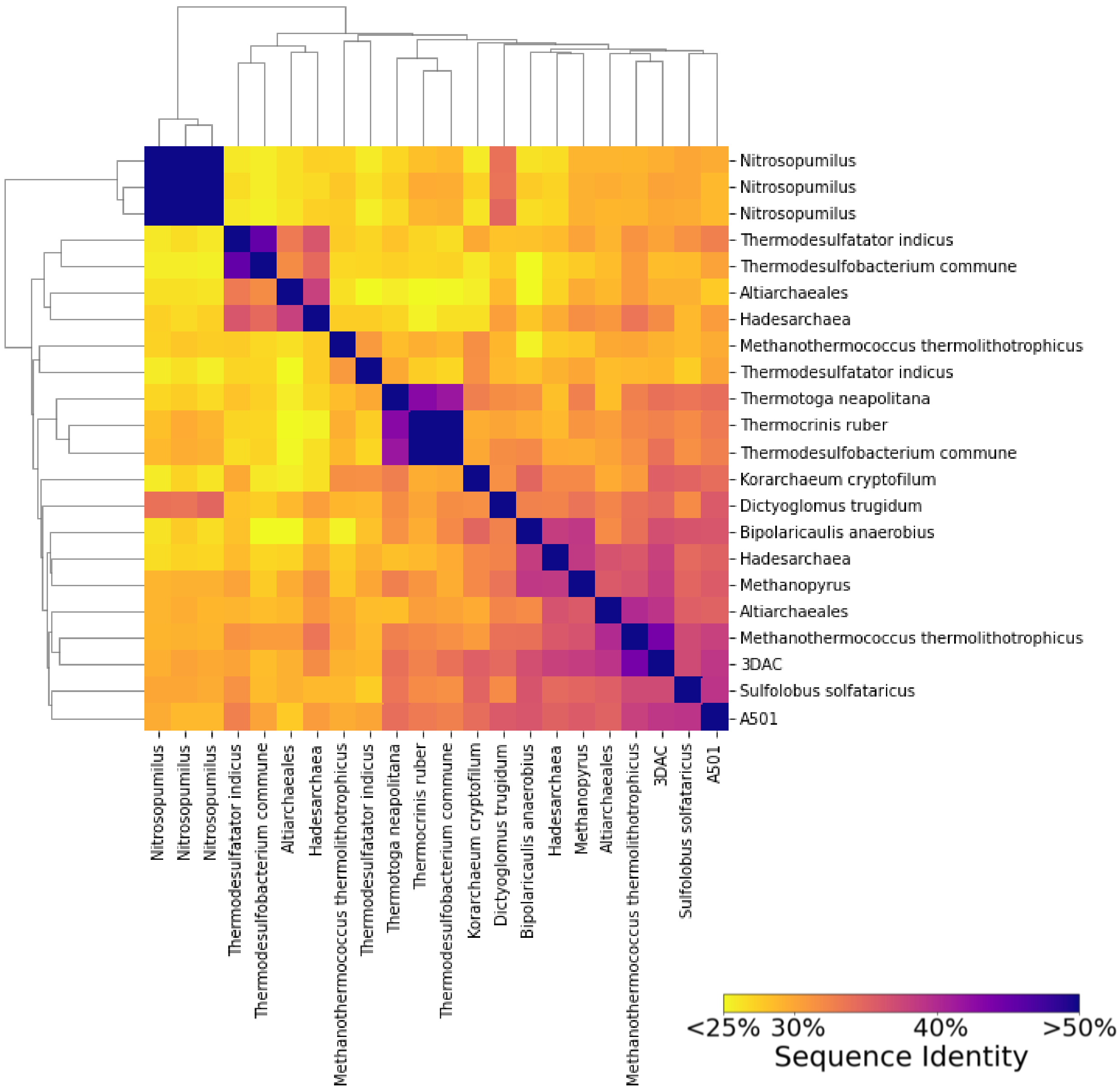

(E) Enolase (EC 4.2.1.11): structure

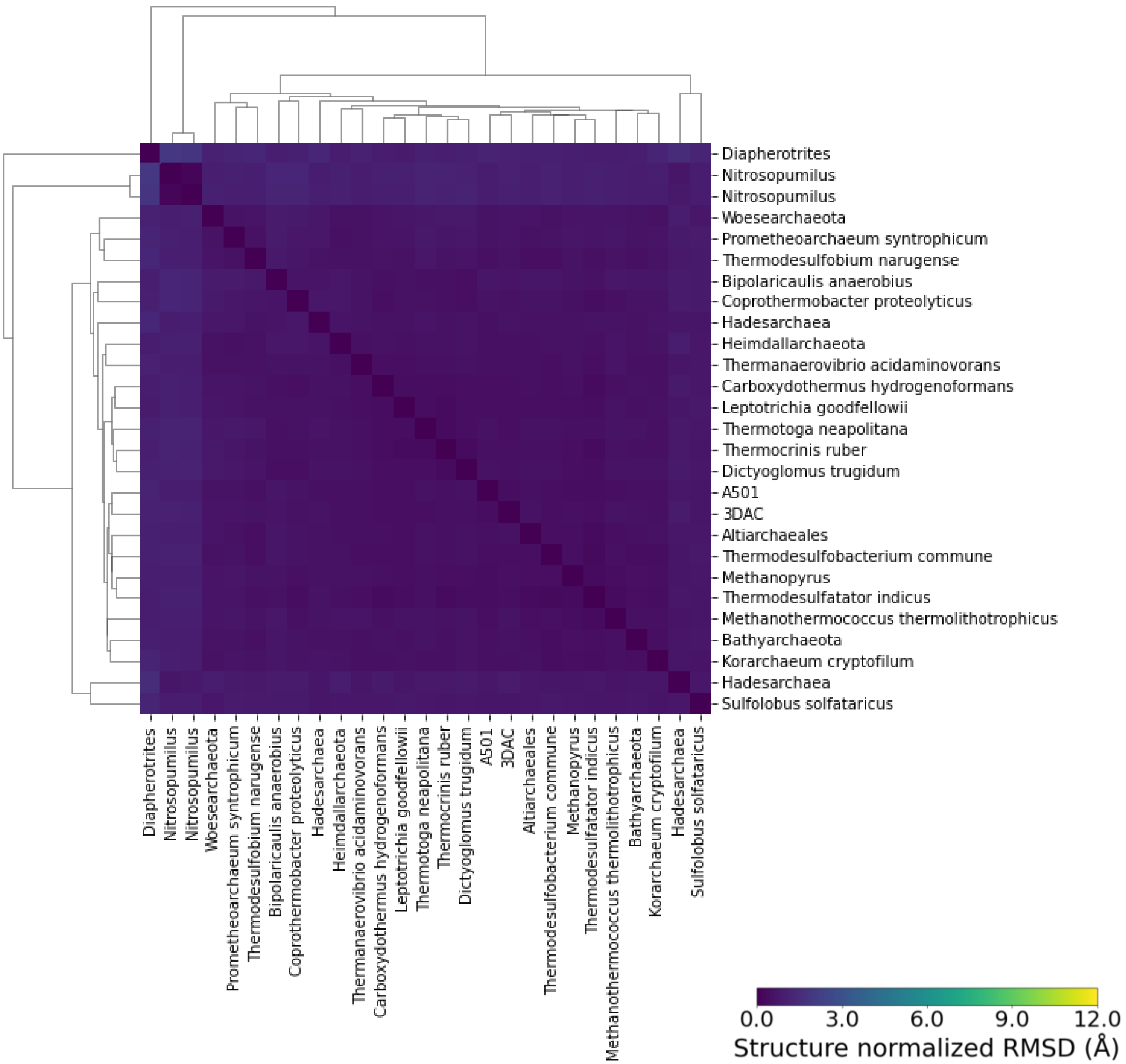

(E) Enolase (EC 4.2.1.11): sequence

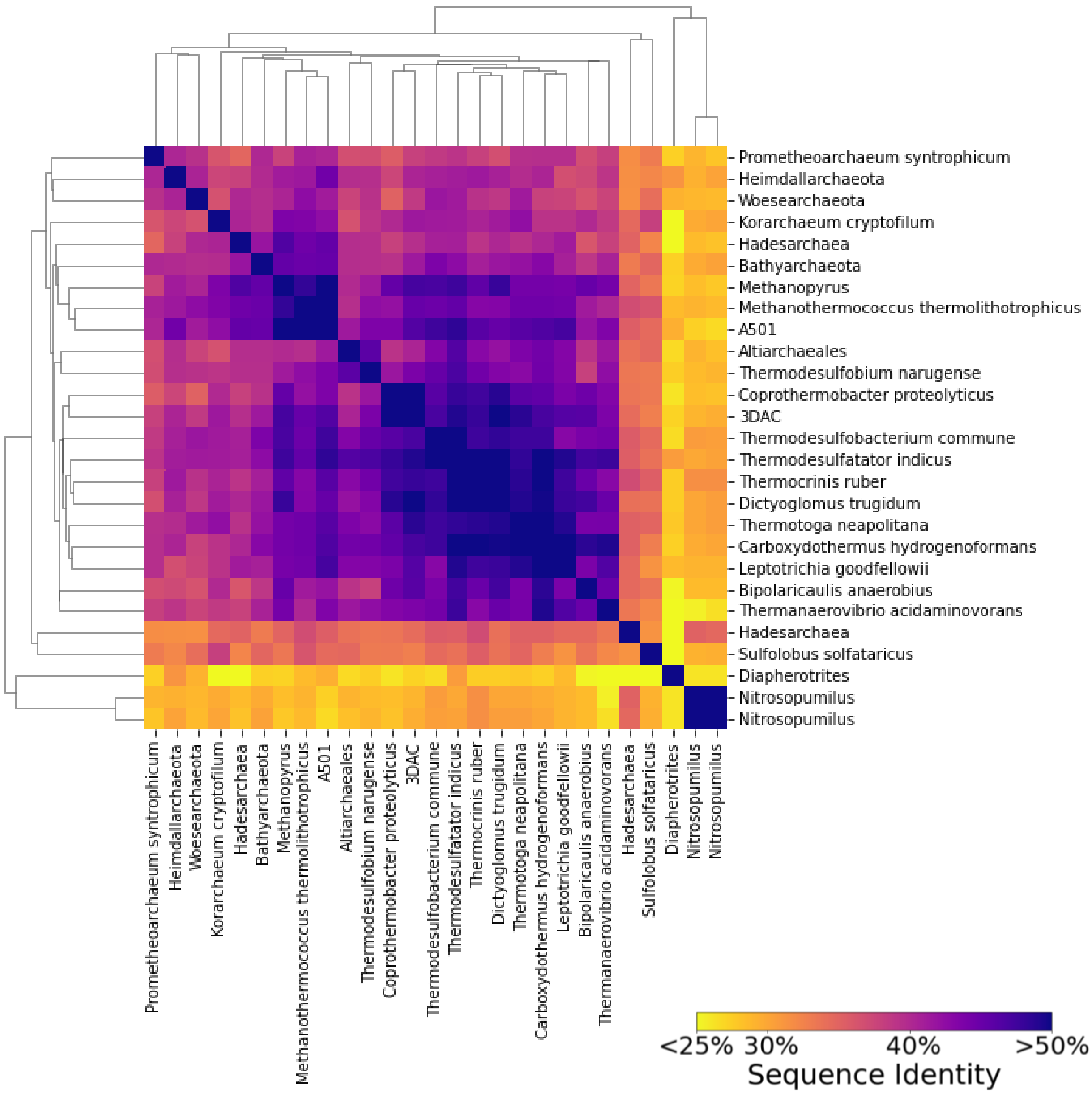

(F) Pyruvate kinase (EC 2.7.1.40): structure

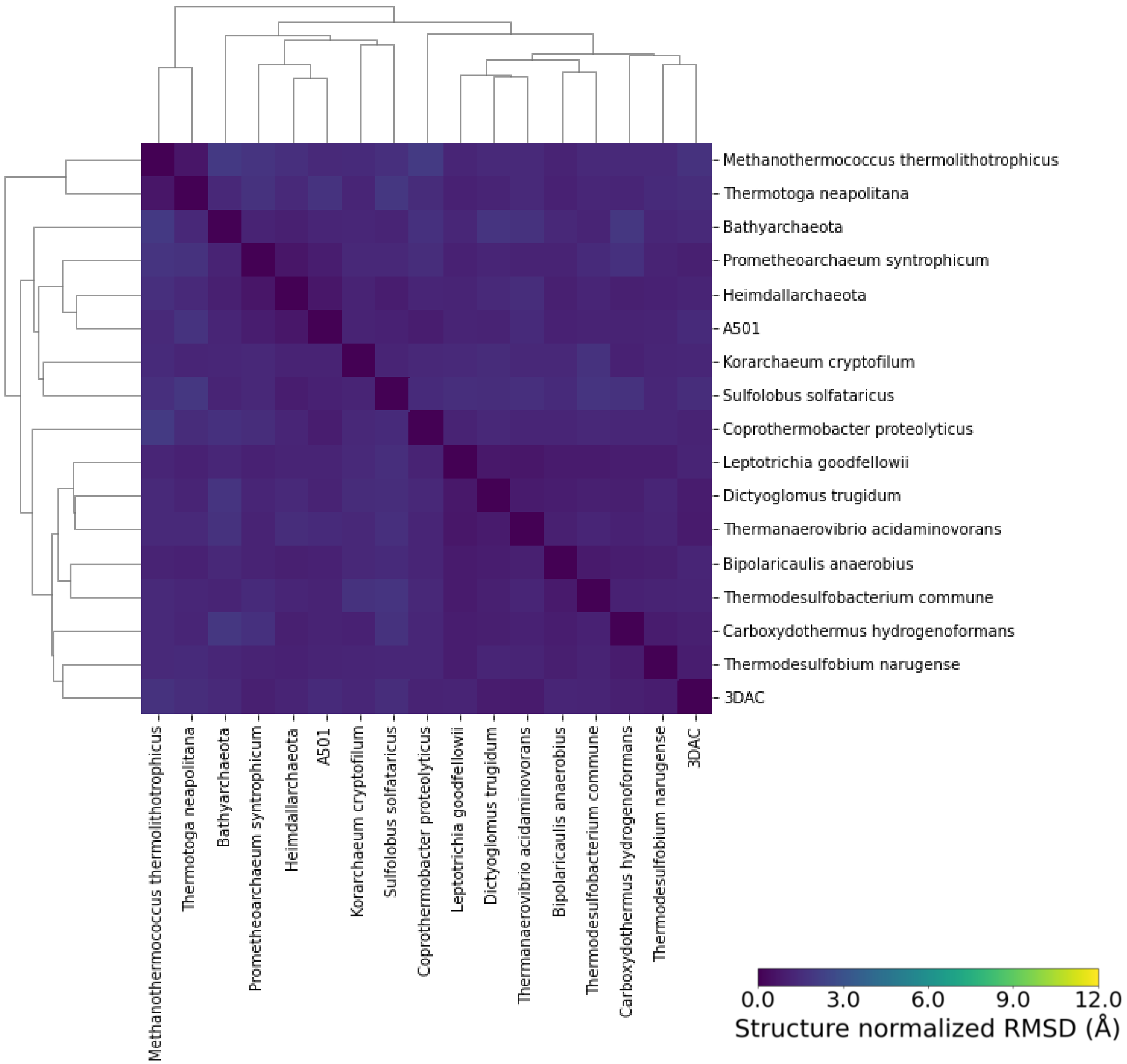

(F) Pyruvate kinase (EC 2.7.1.40): sequence

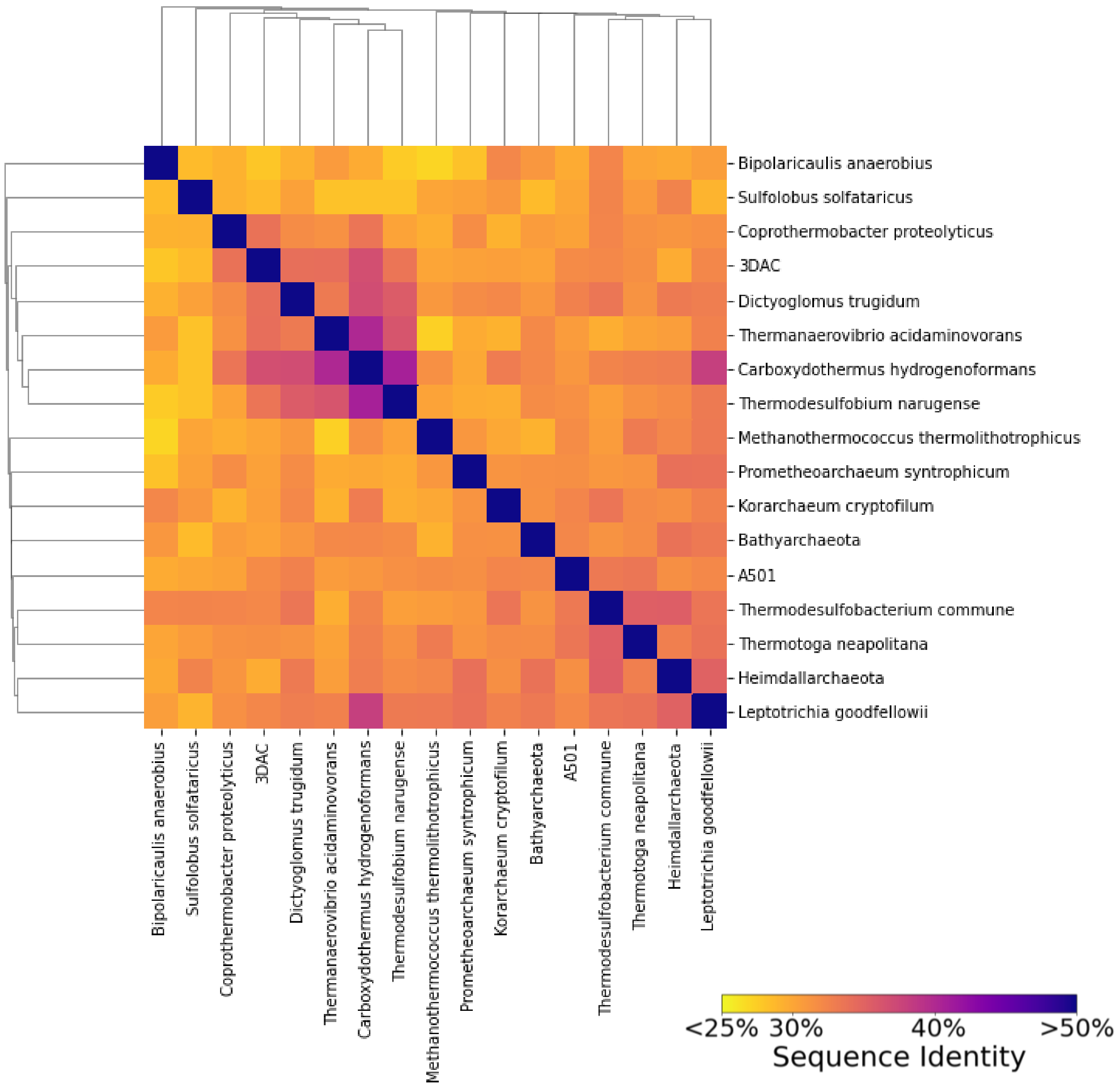

(G) Pyruvate/2-oxoacid oxidoreductase (EC 1.2.7.11): structure

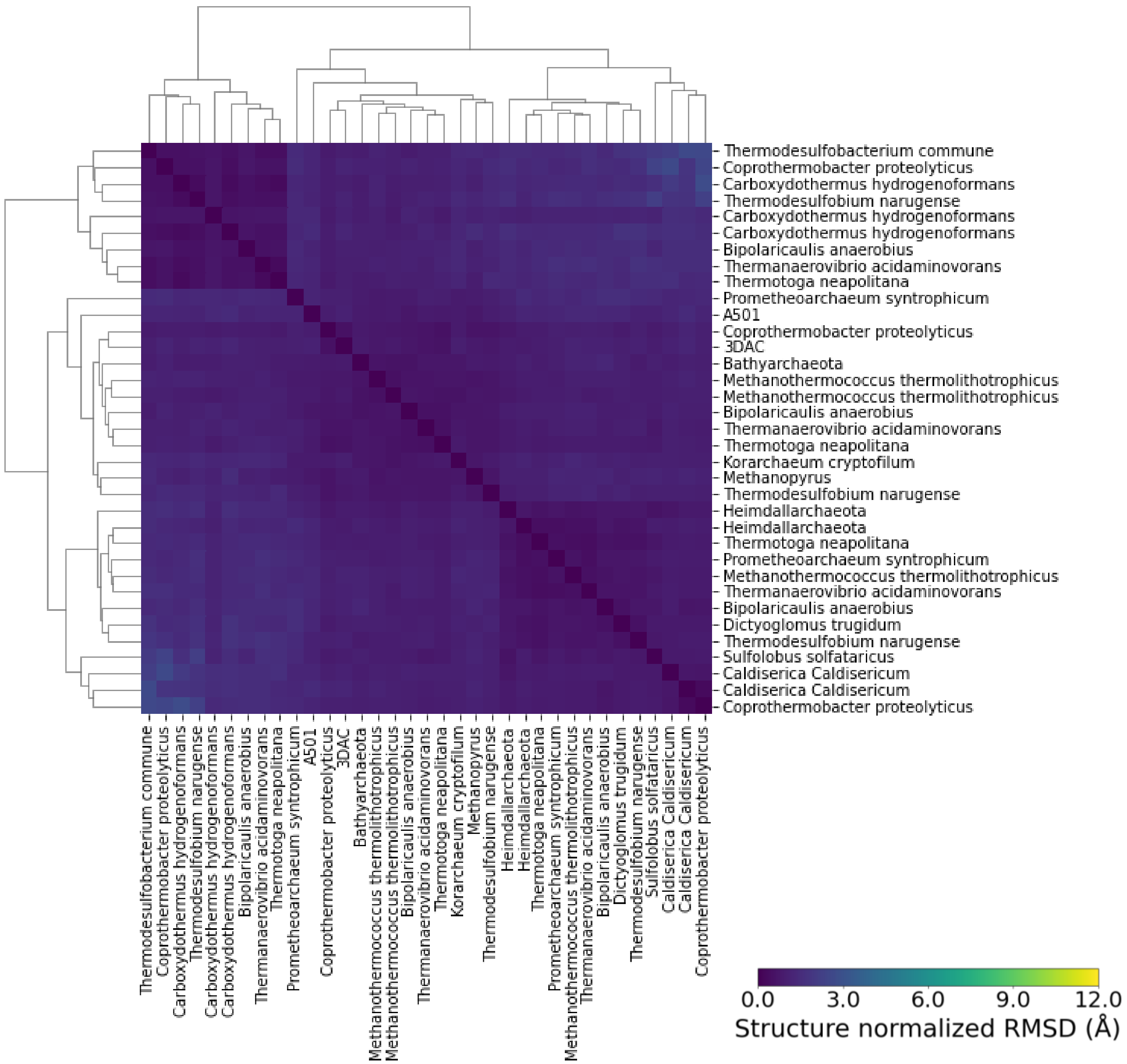

(G) Pyruvate/2-oxoacid oxidoreductase (EC 1.2.7.11): sequence

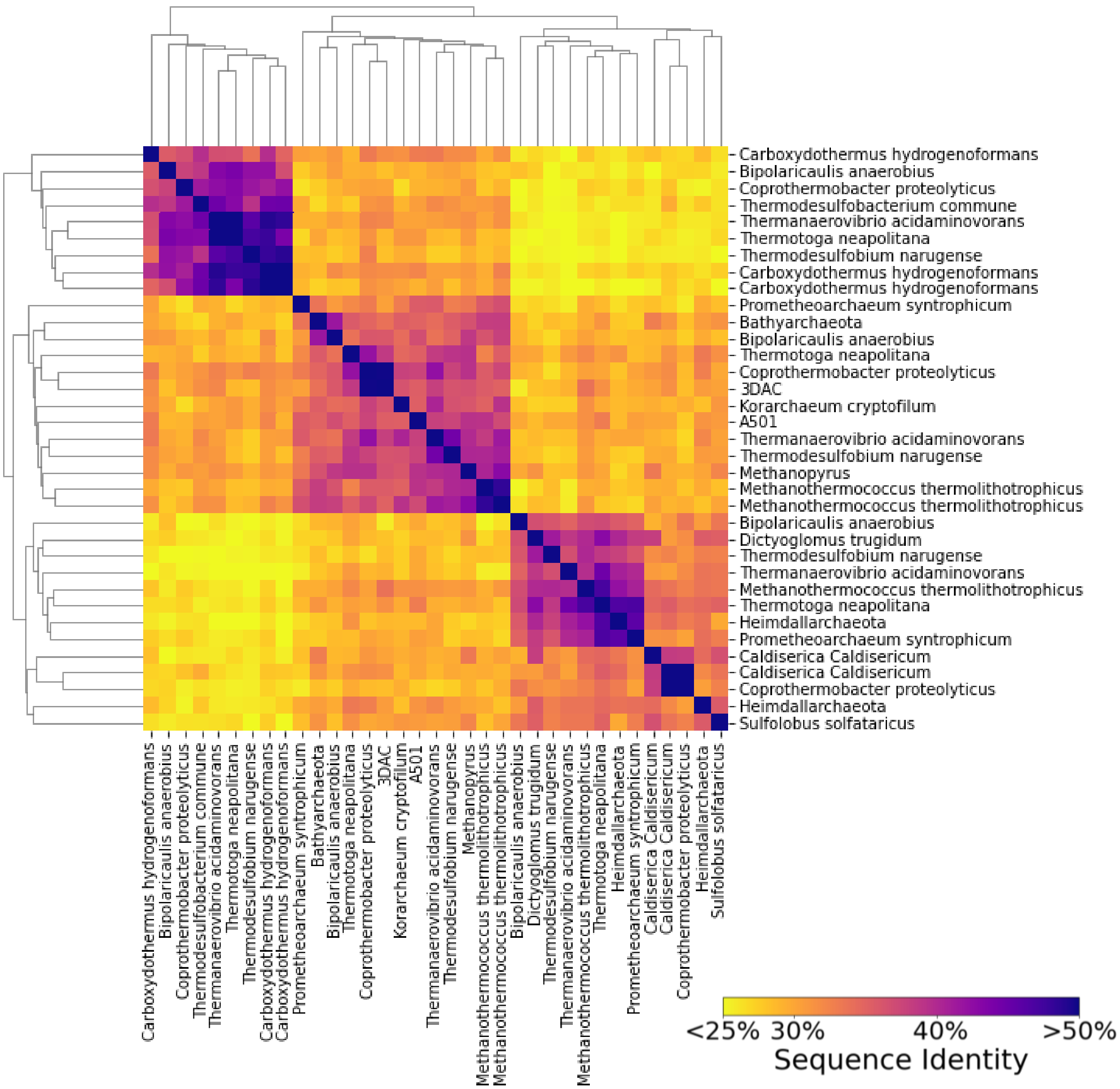

Supplementary Figure 8. AlphaFold-Multimer predicted structures of membrane bound sulfane sulfur reduction complex (MBS) in A501 (A) and 3DAC (B). Subunits in purple represent those proteins with ortholog genes and similar structures, which can be classified as group (i) in the main text, subunits in green represent those proteins with non-ortholog genes but similar structures, which can be classified as group (ii) in the main text, while subunits in white represent those proteins with non-ortholog genes and different structures, which can be classified as group (iii) in the main text. Complex structures were predicted by multimer model in AlphaFold v2.2.[10].

(A) MBS in A501

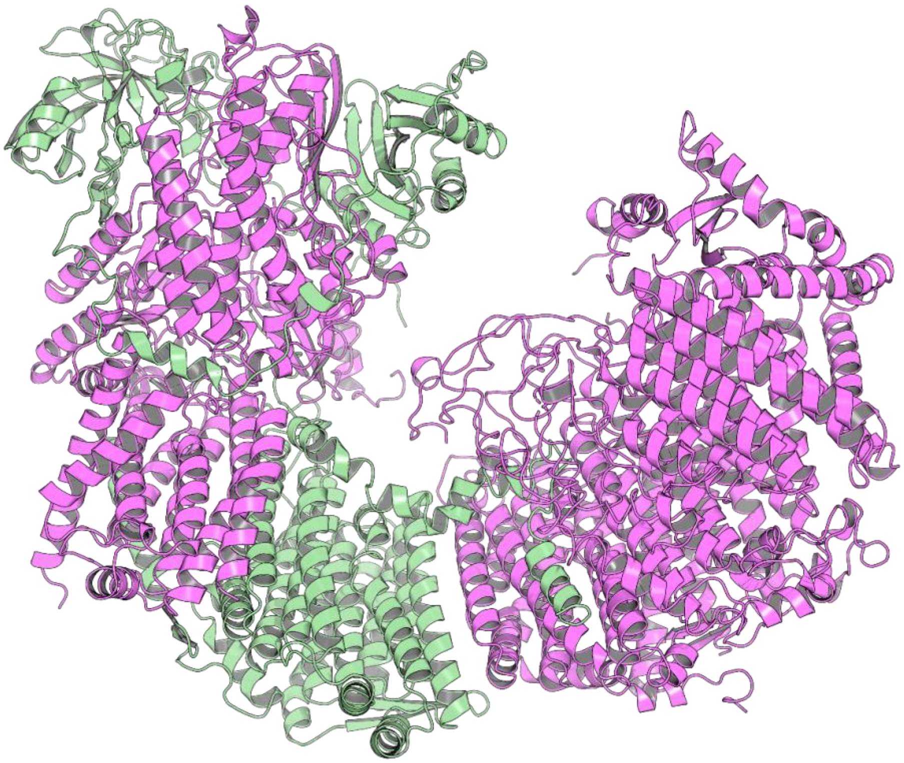

(B) MBS in 3DAC

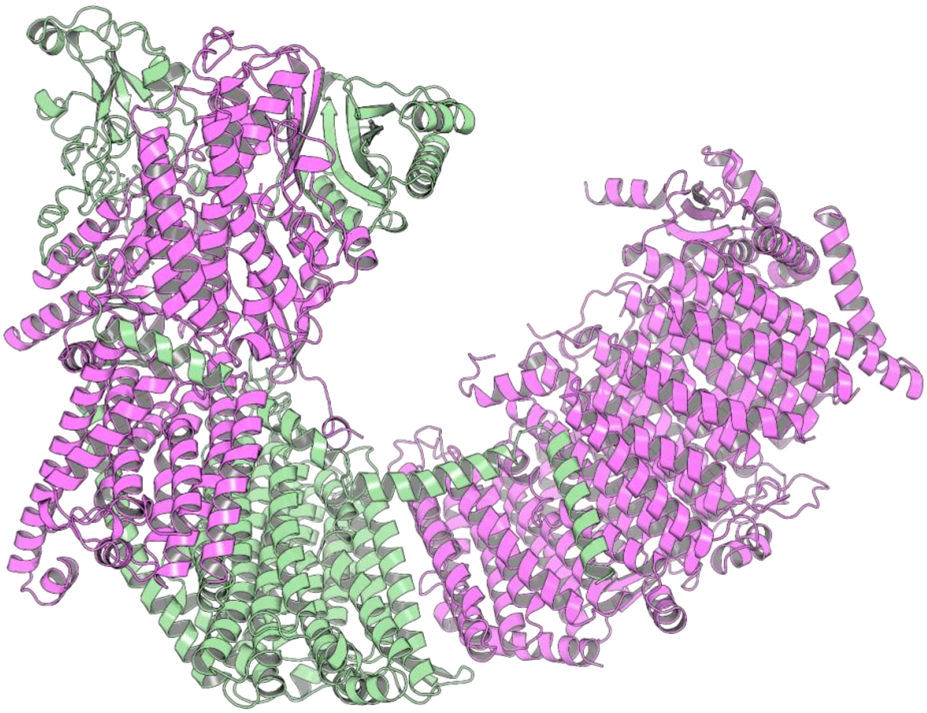

(C) MBH in A501

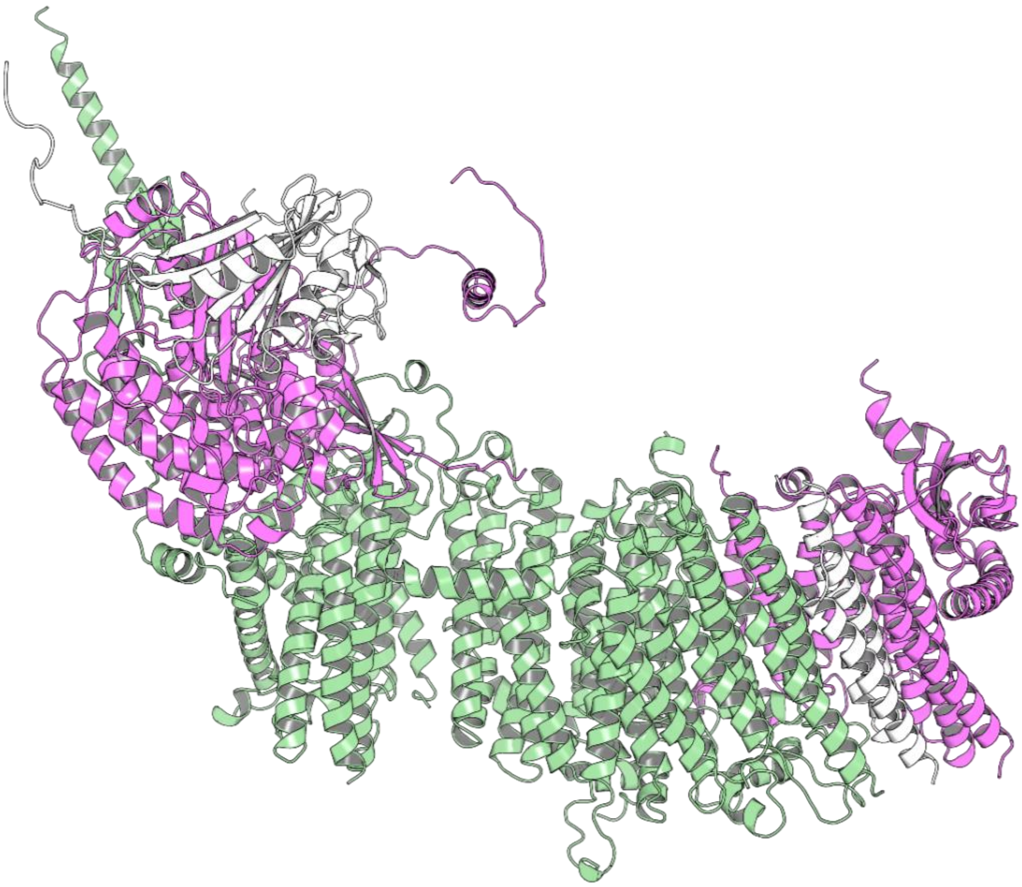

(D) MBH in 3DAC

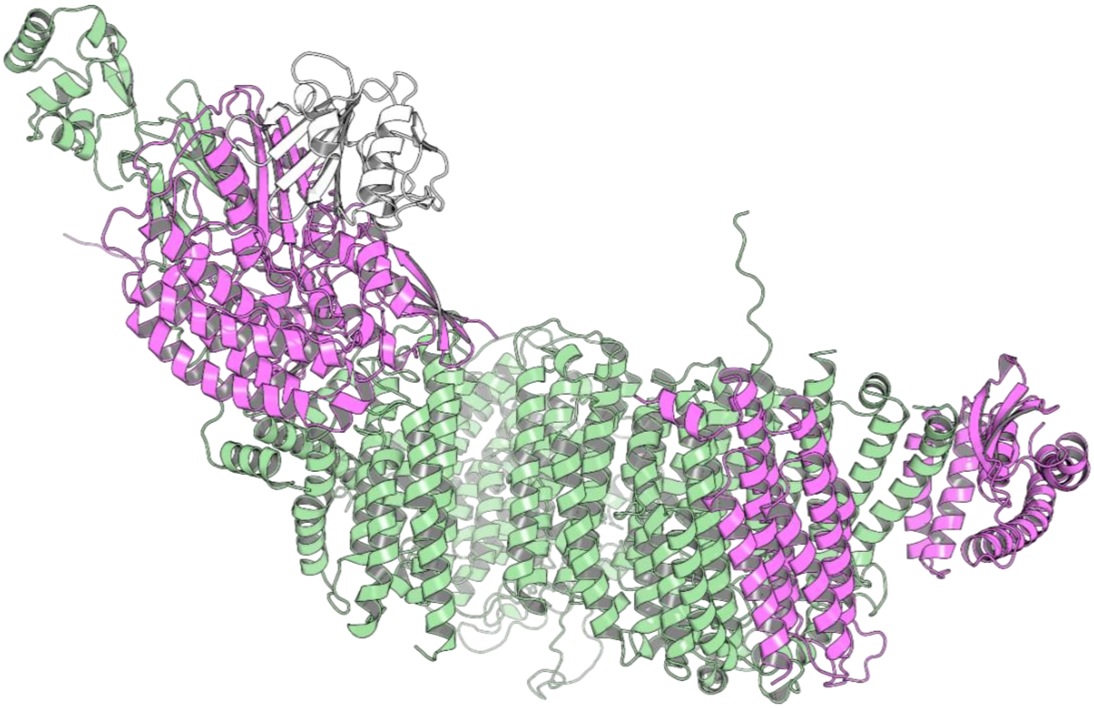

Supplementary Figure 9. Structural properties differences before and after the AlphaFold relax step. The A501 protein structures (n=2,180) are colored in red, while 3DAC structures (n=1,498) are represented in blue. The comparison of properties (by relaxed structure minus unrelaxed structure) includes hydrogen bond per residue **(A)**, salt bridge pre residue **(B)**, RSA **(C)** and disulfide bond **(D)**. After relax, interaction between residues like hydrogen bond, salt bridge and disulfide bond has increased, while RSA has decreased. This may support that the relax step in AlphaFold is crucial for structure analysis. The error bars show the range of distribution.

(A) Difference in hydrogen bond per residue

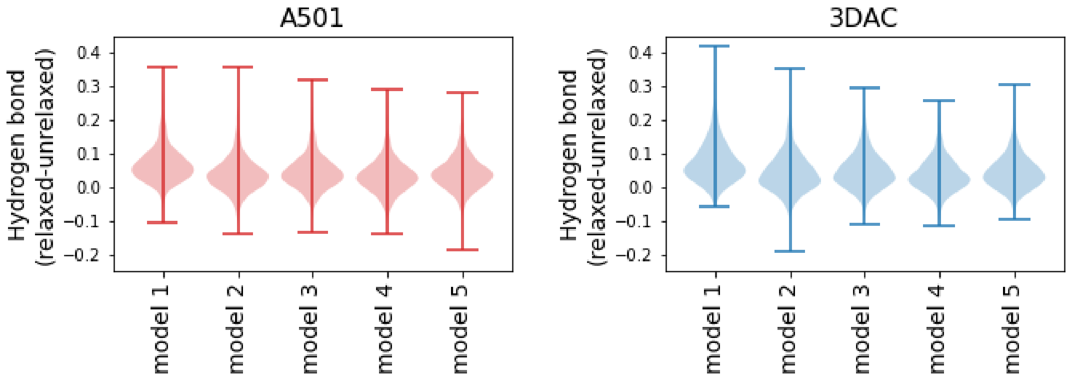

(B) Difference in salt bridge per residue

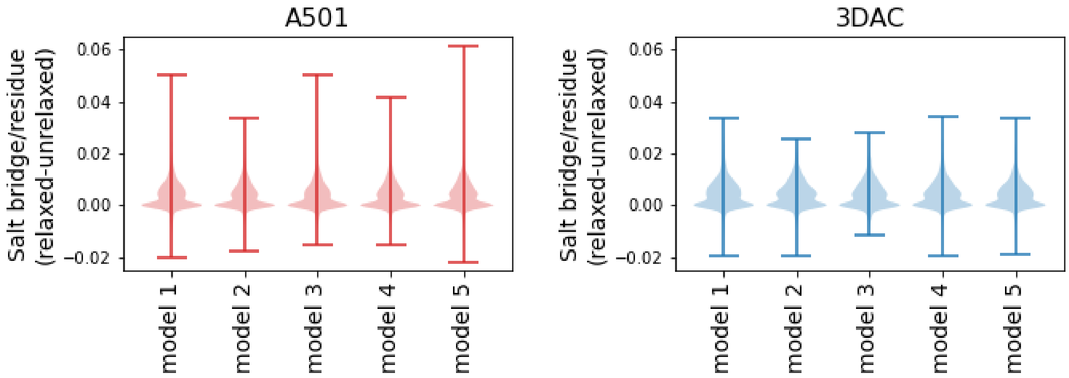

(C) Difference in RSA (relative surface area)

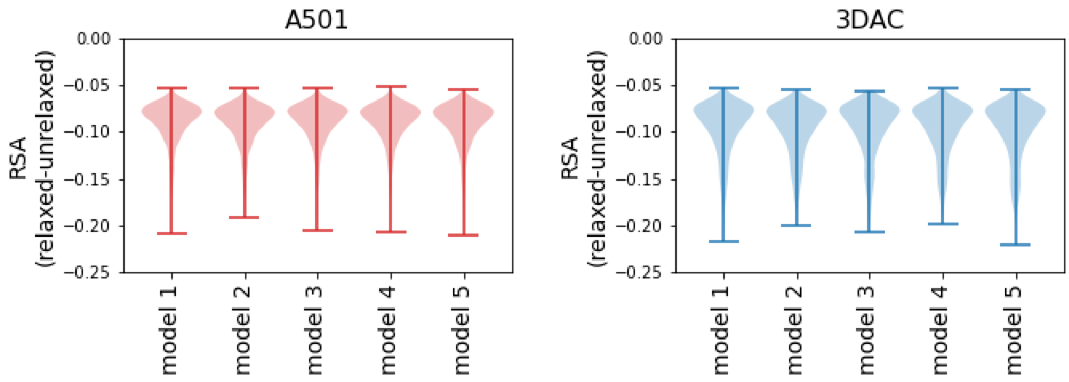

(D) Difference in disulfide bond

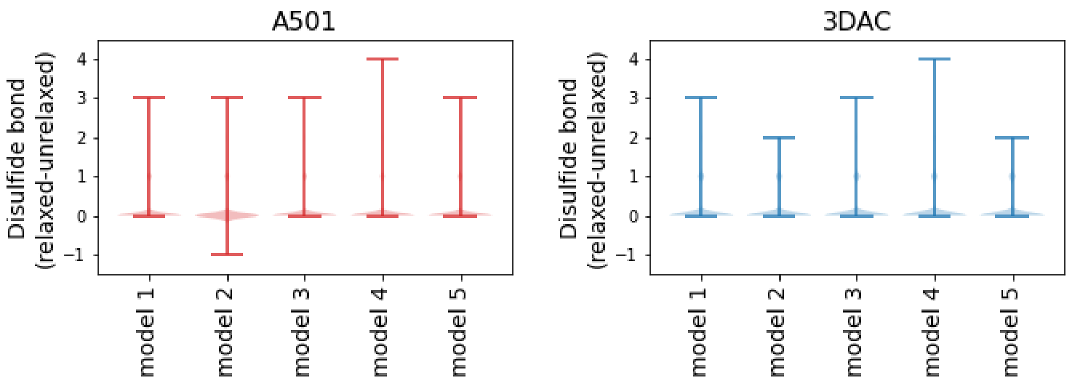

Supplementary Figure 10. Structural analysis between high and low-pLDDT proteins. (A) Compared the correlation between protein length and protein average pLDDT. (B) Compared the correlation between protein loop percentage and protein average pLDDT. (C) presented the distribution of protein length before (A501 n=2,180, 3DAC n=1,498) and after (A501 n=2,022, 3DAC n=1,194) pLDDT-based filter (all prediction models' pLDDT > 70). The Wilcoxon rank sum test between the distribution showed there is no significant difference in protein length (A501 p-value: 0.05307, 3DAC p-value: 0.7248, 2-sided Wilcoxon rank sum test). The error bands show the confidence interval for the regression estimate. The error bar in (C) shows the range of distribution.

(A) Correlation between protein length and protein average pLDDT

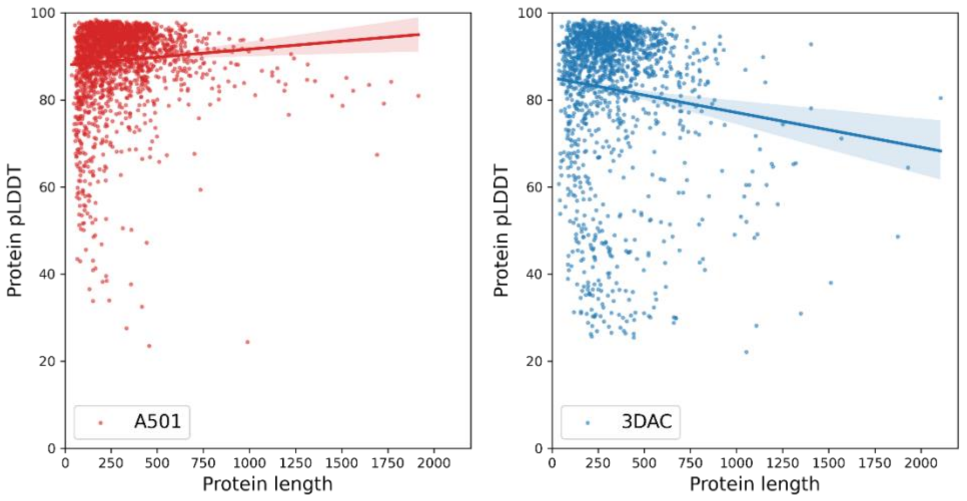

(B) Correlation between protein loop percentage and protein average pLDDT

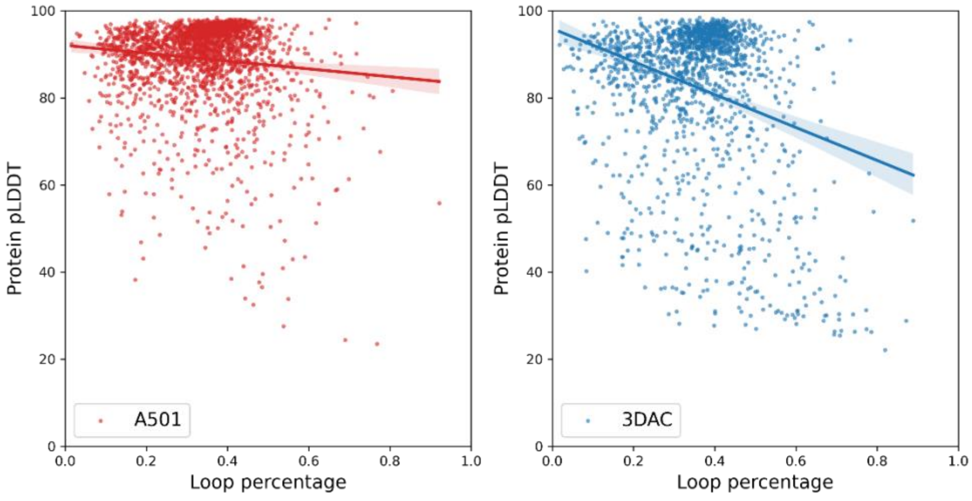

(C) Protein length distribution differences before and after pLDDT-filter

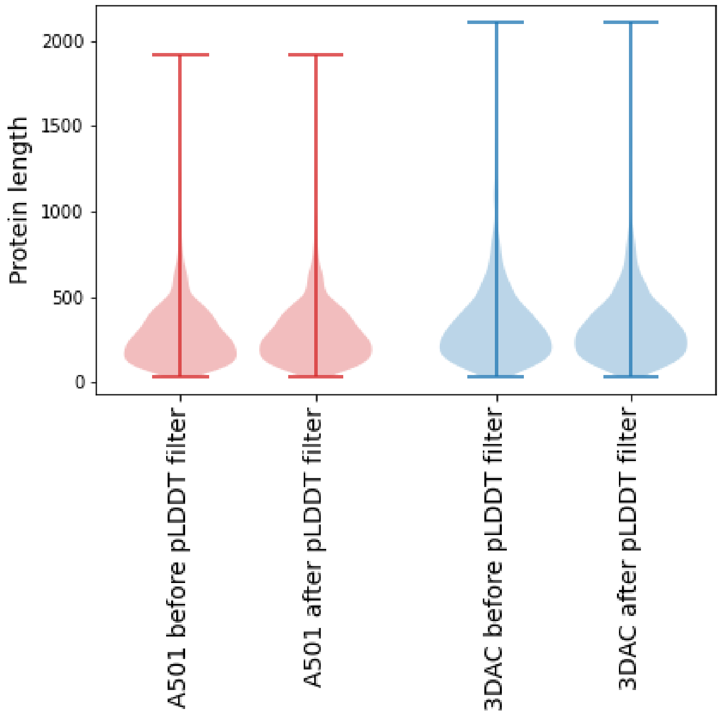

Supplementary Figure 11. Chirality analysis of Cα in all AlphaFold predicted structures in A501 **(A, B)** and 3DAC **(C, D)**. Consider all residues Cα(except glycine) should adopt a left handed conformation, the wrong chirality count in prediction set and wrong chirality rate is shown in this figure. We also compared the protein structure set before and after pLDDT filter (all models pLDDT > 70)

(A) Wrong chirality count and rate in all predicted A501 structures

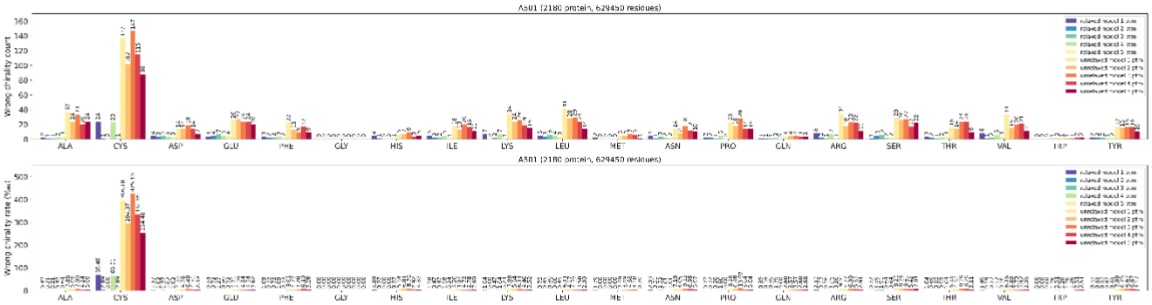

(B) Wrong chirality count and rate in pLDDT-filtered A501 structures

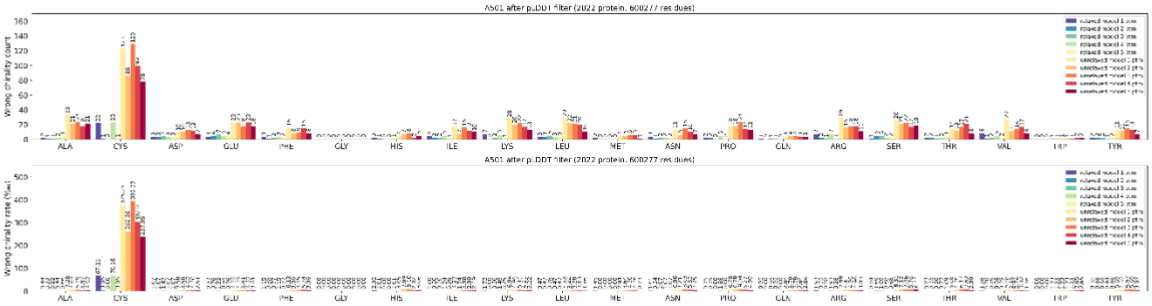

(C) Wrong chirality count and rate in all predicted 3DAC structures

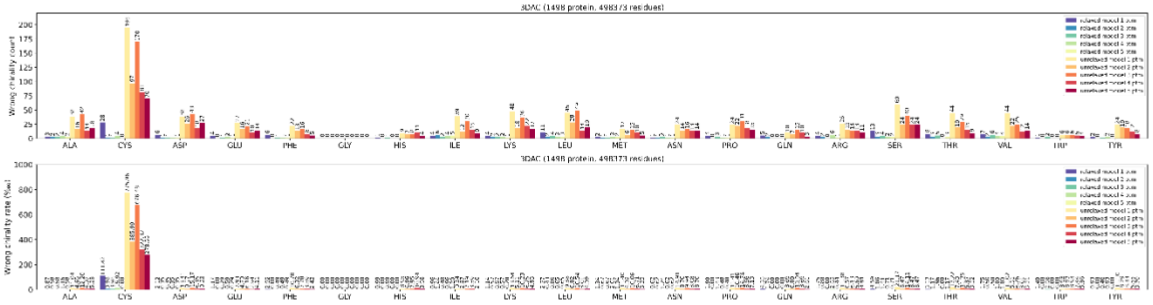

(D) Wrong chirality count and rate in pLDDT-filtered 3DAC structures

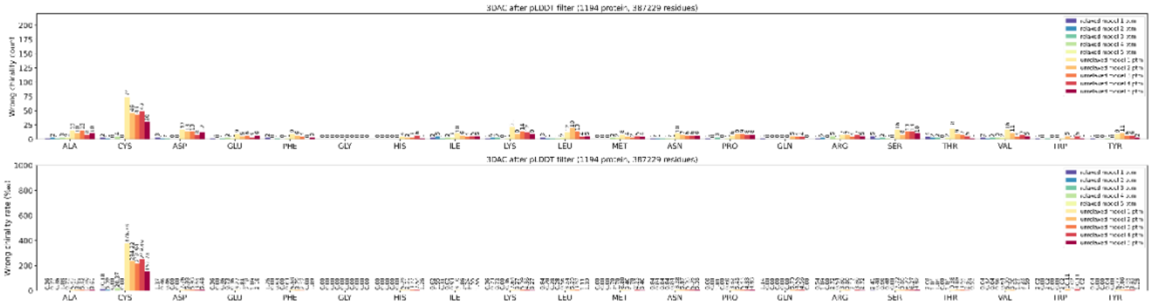

Supplementary Figure 12. Cis peptide bond rate analysis (ω-dihedral angel) in all AlphaFold predicted structures in A501 **(A)** and 3DAC **(B)**. Peptide bonds are classified into 4 categories by its neighbor residues, "Pro" corresponds to proline, and "Xaa" corresponds to all other amino acids.

(A) Cis-peptide bond rate of all predicted A501 structures

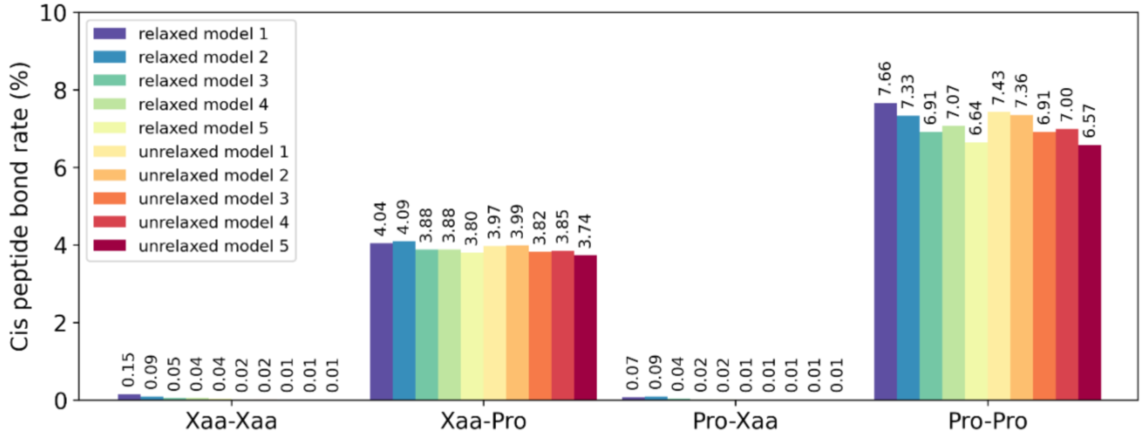

(B) Cis-peptide bond rate of all predicted 3DAC structures

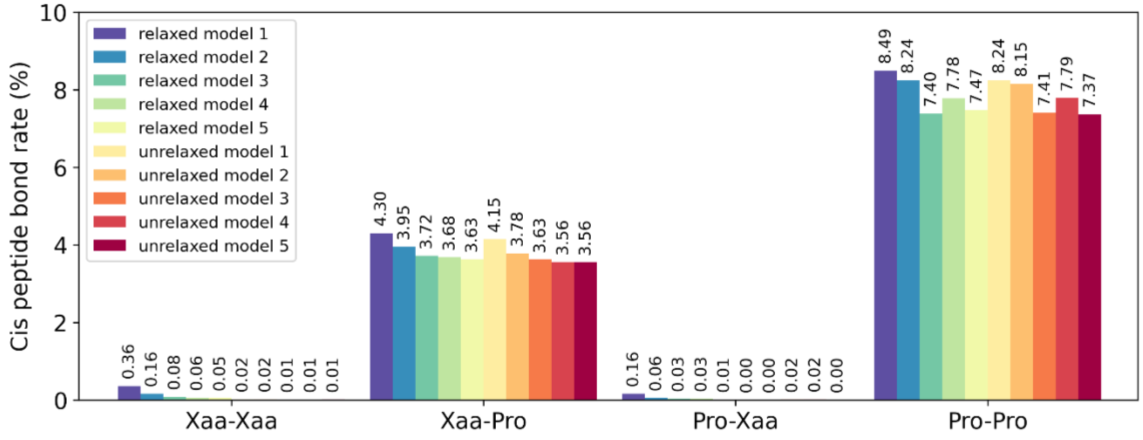

Supplementary Figure 13. Distribution of protein pairs analyzed with functions and classified into group (i) and (ii) and (iii) in this study. The dashed lines represent the threshold used in this study.

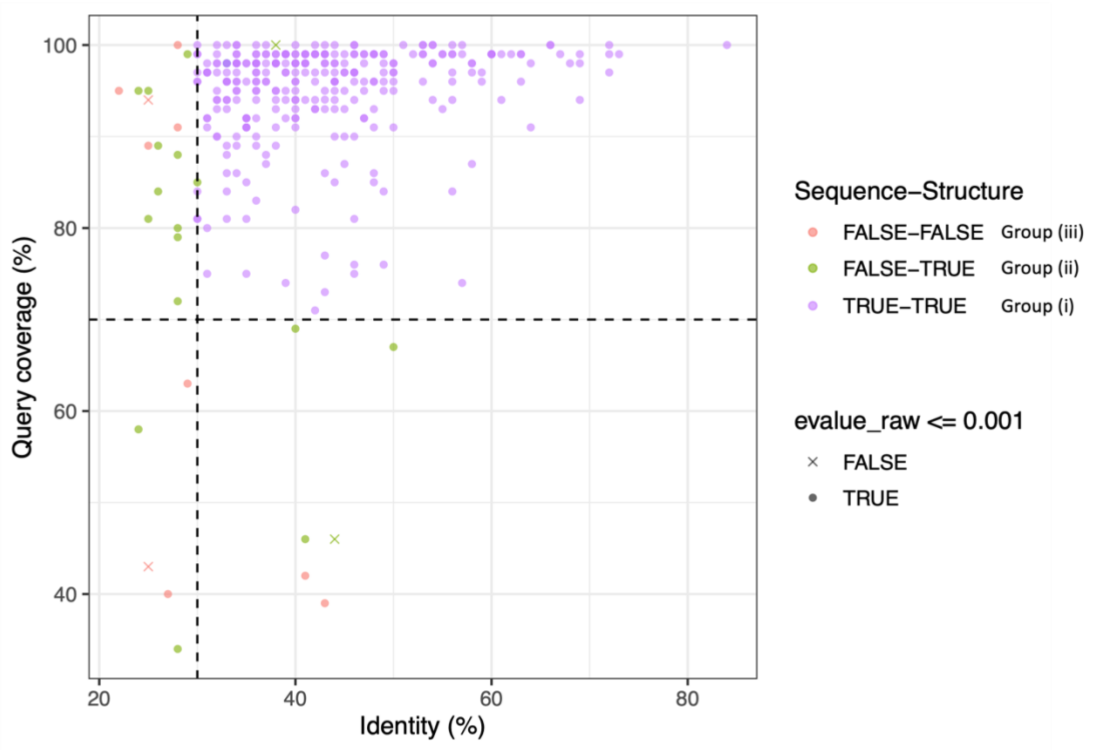

Supplementary Data

Supplementary Data 1. Comparison of structures of each protein pair between A501 and 3DAC.

Supplementary Data 2. Detailed information for the comparison of seven key enzymes in the central carbon metabolism among extended 12 archaeal and 12 bacterial strains.

SI References:

1. Amend, J. P. & Shock, E. L. Energetics of amino acid synthesis in hydrothermal ecosystems. *Science* **281**, 1659-1662 (1998).
2. Menez, B. *et al.* Abiotic synthesis of amino acids in the recesses of the oceanic lithosphere. *Nature* **564**, 59-63 (2018).
3. Patel, B. H., Percivalle, C., Ritson, D. J., Duffy, C. D. & Sutherland, J. D. Common origins of RNA, protein and lipid precursors in a cyanosulfidic protometabolism. *Nat. Chem.* **7**, 301-307 (2015).
4. Frenkel-Pinter, M. *et al.* Selective incorporation of proteinaceous over nonproteinaceous cationic amino acids in model prebiotic oligomerization reactions. *Proc. Natl. Acad. Sci. U.S.A.* **116**, 16338-16346 (2019).
5. Tehei, M. *et al.* Adaptation to extreme environments: macromolecular dynamics in bacteria compared in vivo by neutron scattering. *EMBO Rep.* **5**, 66-70 (2004).
6. Jasnin, M., Tehei, M., Moulin, M., Haertlein, M. & Zaccai, G. Solvent isotope effect on macromolecular dynamics in *E.coli*. *Eur. Biophys. J. Biophy.* **37**, 613-617 (2008).
7. Weiss, M. S., Jabs, A. & Hilgenfeld, R. Peptide bonds revisited. *Nat. Struct. Biol.* **5**, 676-676 (1998).
8. Zhang, Y. & Sievert, S. M. Pan-genome analyses identify lineage- and niche-specific markers of evolution and adaptation in Epsilonproteobacteria. *Front. Microbiol.* **5** (2014).
9. Pearson, W. R. An introduction to sequence similarity ("homology") searching. *Curr. Protoc. Bioinform.* **42**, 1-8 (2013).

10. Evans, R. *et al.* Protein complex prediction with AlphaFold-Multimer. Preprint at https://www.biorxiv.org/content/10.1101/2021.10.04.463034v2 (2022).

11. Zhao, W. & Zhong, B. Proteome-wide structure prediction of A501 and 3DAC proteome by AlphaFold. https://doi.org/10.5281/zenodo.6300206 (2022)

12. Zhao, W. & Zhong, B. Predicted structure database of 7 key enzymes in the central carbon metabolism among extended 12 archaeal and 12 bacterial strains. https:/doi.org/10.5281/zenodo.6387901 (2022).
