# Supplementary material for: Proteome-wide 3D structure prediction provides insights into the ancestral metabolism of ancient archaea and bacteria
Source: Nat Commun. 2022 Dec 21;13:7861. doi: 10.1038/s41467-022-35523-8 (PMC9772386; doi:10.1038/s41467-022-35523-8)
Supplement: Supplementary file 3 — Description of Additional Supplementary Files [file 41467_2022_35523_MOESM3_ESM.pdf]

Description of Additional Supplementary Files

Supplementary Data 1: Comparison of structures of each protein pair between A501 and 3DAC.

Supplementary Data 2: Detailed information for the comparison of seven key enzymes in the central carbon metabolism among extended 12 archaeal and 12 bacterial strains.
