# Supplementary material for: Proteome-wide 3D structure prediction provides insights into the ancestral metabolism of ancient archaea and bacteria
Source: Nat Commun. 2022 Dec 21;13:7861. doi: 10.1038/s41467-022-35523-8 (PMC9772386; doi:10.1038/s41467-022-35523-8)
Supplement: Supplementary file 6 — Reporting Summary [file 41467_2022_35523_MOESM6_ESM.pdf]

nature portfolio

Corresponding author(s): Xiang Xiao and Liang Hong

Last updated by author(s): Nov 25, 2022

Reporting Summary

Nature Portfolio wishes to improve the reproducibility of the work that we publish. This form provides structure for consistency and transparency in reporting. For further information on Nature Portfolio policies, see our Editorial Policies and the Editorial Policy Checklist.

Statistics

For all statistical analyses, confirm that the following items are present in the figure legend, table legend, main text, or Methods section.

n/a	Confirmed	
☐	☒	The exact sample size (*n*) for each experimental group/condition, given as a discrete number and unit of measurement
☐	☒	A statement on whether measurements were taken from distinct samples or whether the same sample was measured repeatedly
☐	☒	The statistical test(s) used AND whether they are one- or two-sided<br>*Only common tests should be described solely by name; describe more complex techniques in the Methods section.*
☐	☒	A description of all covariates tested
☐	☒	A description of any assumptions or corrections, such as tests of normality and adjustment for multiple comparisons
☐	☒	A full description of the statistical parameters including central tendency (e.g. means) or other basic estimates (e.g. regression coefficient) AND variation (e.g. standard deviation) or associated estimates of uncertainty (e.g. confidence intervals)
☐	☒	For null hypothesis testing, the test statistic (e.g. *F*, *t*, *r*) with confidence intervals, effect sizes, degrees of freedom and *P* value noted<br>*Give P values as exact values whenever suitable.*
☒	☐	For Bayesian analysis, information on the choice of priors and Markov chain Monte Carlo settings
☒	☐	For hierarchical and complex designs, identification of the appropriate level for tests and full reporting of outcomes
☐	☒	Estimates of effect sizes (e.g. Cohen's *d*, Pearson's *r*), indicating how they were calculated

Our web collection on statistics for biologists contains articles on many of the points above.

Software and code

Policy information about availability of computer code

Data collection	Data Collection:<br>The complete genome sequences of Thermococcus eurythermalis A501 (NCBI accession: CP008887.1) and Zhurongbacter thermophilus 3DAC (NCBI accession: CP046447).<br>The structure data of corresbonding genome sequences are predicted by open source software ParaFold based on AlphaFold. ParaFold (v1.0) has identical neural network and multiple sequence alignment method with AlphaFold (v.2.1.1), only has a optimized pipeline to accelerate proteome-wide structure prediction. For each predicted protein, we used the same settings (used templates before 2021-07-27, actually the template dataset collected templates before 2020-05-14).<br>The structure data from other model species are downloaded from AlphaFold database https://alphafold.ebi.ac.uk/ under a CC-BY-4.0 licence.
Data analysis	The analysis of the data were performed with licenced or open-sourced software. They are:<br>PyMOL v2.0 under academic license https://pymol.org/<br>biopython: https://biopython.org/<br>MAFFT algorithm v7.313<br>All original codes involved in this manuscript are available in GitHub repository: https://github.com/weishuzhao/A501-3DAC-AlphaFold.

For manuscripts utilizing custom algorithms or software that are central to the research but not yet described in published literature, software must be made available to editors and reviewers. We strongly encourage code deposition in a community repository (e.g. GitHub). See the Nature Portfolio guidelines for submitting code & software for further information.

Data

Policy information about availability of data

All manuscripts must include a data availability statement. This statement should provide the following information, where applicable:

- Accession codes, unique identifiers, or web links for publicly available datasets
- A description of any restrictions on data availability
- For clinical datasets or third party data, please ensure that the statement adheres to our policy

Data availability
All source data are provided with this paper.
The complete genome sequences of Thermococcus eurythermalis A501 (NCBI accession: CP008887.1) and Zhurongbacter thermophilus 3DAC (NCBI accession: CP046447) are available at https://www.ncbi.nlm.nih.gov/
ParaFold (v1.0) is used for proteome-wide structure prediction, which has identical neural network and multiple sequence alignment method with AlphaFold (v.2.1.1).
The proteome-wide structure predictions of Thermococcus eurythermalis A501 and Zhurongbacter thermophilus 3DAC are available at: https://zenodo.org/record/6300206.
Protein structure predictions for 155 additional proteins of 24 additional archaeal and bacterial species are available at: https://zenodo.org/record/6387901.
The structure data from other model species are downloaded from AlphaFold database https://alphafold.ebi.ac.uk/ under a CC-BY-4.0 license.
PDB entries used in this study can be found in https://www.rcsb.org/.

Code availability
The code for processing, analyzing and visualizing the results is available at: https://github.com/weishuzhao/A501-3DAC-AlphaFold.
ParaFold to conduct proteome-wide structure prediction based on AlphaFold pipeline is an open-source software (MIT) available at https://github.com/Zuricho/ParallelFold.

Human research participants

Policy information about studies involving human research participants and Sex and Gender in Research.

Reporting on sex and gender	Not applicable
Population characteristics	Not applicable
Recruitment	Not applicable
Ethics oversight	Not applicable

Note that full information on the approval of the study protocol must also be provided in the manuscript.

Field-specific reporting

Please select the one below that is the best fit for your research. If you are not sure, read the appropriate sections before making your selection.

☒ Life sciences ☐ Behavioural & social sciences ☐ Ecological, evolutionary & environmental sciences

For a reference copy of the document with all sections, see nature.com/documents/nr-reporting-summary-flat.pdf

Life sciences study design

All studies must disclose on these points even when the disclosure is negative.

Sample size	We used the proteome-wide structure predictions of two strains in this study. The choose of sample size is based on all proteins encoded by the complete genome of these two strains.
Data exclusions	The confidence of the accuracy of the predicted structures can be measured by the predicted local distance difference test (pLDDT) using the lDDT-Cα metric. Residues with pLDDT score ≥ 70 are classified as high-confidence residues, which means that at least 70% of local Cα distances (local means distance < 15 Å) have an error within 4 Å. So we excluded predicted protein structures of those pLDDT < 70 in this study, and only keep the predicted structure with high confidence (pLDDT > 70).
Replication	All physiological and metabolic experiments are in independent triplicates in this study. All replications performed successfully and used in the statistic analysis in this study.
Randomization	All experiments performed in this study allocated samples or replicates into experimental groups with randomization.

Blinding	The investigators were blinded to group allocation during data collection and/or analysis in all experiments performed in this study.

Reporting for specific materials, systems and methods

We require information from authors about some types of materials, experimental systems and methods used in many studies. Here, indicate whether each material, system or method listed is relevant to your study. If you are not sure if a list item applies to your research, read the appropriate section before selecting a response.

Materials & experimental systems

n/a	Involved in the study	
☒	☐	Antibodies
☒	☐	Eukaryotic cell lines
☒	☐	Palaeontology and archaeology
☒	☐	Animals and other organisms
☒	☐	Clinical data
☒	☐	Dual use research of concern

Methods

n/a	Involved in the study	
☒	☐	ChIP-seq
☒	☐	Flow cytometry
☒	☐	MRI-based neuroimaging
